# Rare-earth-based chalcogenides and their derivatives: an encouraging IR nonlinear optical material candidate

**DOI:** 10.1039/d4sc00697f

**Published:** 2024-03-22

**Authors:** Ping Feng, Jia-Xiang Zhang, Mao-Yin Ran, Xin-Tao Wu, Hua Lin, Qi-Long Zhu

**Affiliations:** a State Key Laboratory of Structural Chemistry, Fujian Institute of Research on the Structure of Matter, Chinese Academy of Sciences Fuzhou Fujian 350002 China linhua@fjirsm.ac.cn qlzhu@fjirsm.ac.cn; b Fujian Science & Technology Innovation Laboratory for Optoelectronic Information of China Fujian 350108 China; c College of Chemistry, Fuzhou University Fuzhou 350002 China; d Fujian College, University of Chinese Academy of Sciences Fuzhou 350002 China; e University of Chinese Academy of Sciences Beijing 100049 China; f Fujian Key Laboratory of Rare-earth Functional Materials, Fujian Shanhai Collaborative Innovation Center of Rare-earth Functional Materials Longyan 366300 China

## Abstract

With the continuous development of laser technology and the increasing demand for lasers of different frequencies in the infrared (IR) spectrum, research on infrared nonlinear optical (NLO) crystals has garnered growing attention. Currently, the three main commercially available types of borate materials each have their drawbacks, which limit their applications in various areas. Rare-earth (RE)-based chalcogenide compounds, characterized by the unique f-electron configuration, strong positive charges, and high coordination numbers of RE cations, often exhibit distinctive optical responses. In the field of IR-NLO crystals, they have a research history spanning several decades, with increasing interest. However, there is currently no comprehensive review summarizing and analyzing these promising compounds. In this review, we categorize 85 representative examples out of more than 400 non-centrosymmetric (NCS) compounds into four classes based on the connection of different asymmetric building motifs: (1) RE-based chalcogenides containing tetrahedral motifs; (2) RE-based chalcogenides containing lone-pair-electron motifs; (3) RE-based chalcogenides containing [BS_3_] and [P_2_Q_6_] motifs; and (4) RE-based chalcohalides and oxychalcogenides. We provide detailed discussions on their synthesis methods, structures, optical properties, and structure–performance relationships. Finally, we present several favorable suggestions to further explore RE-based chalcogenide compounds. These suggestions aim to approach these compounds from a new perspective in the field of structural chemistry and potentially uncover hidden treasures within the extensive accumulation of previous research.

## Abstract 2

Non-centrosymmetric rare-earth-based chalcogenides and their derivatives could offer novel insights into the targeted design and exploratory synthesis of new IR nonlinear optical candidates.

## Graphical abstract

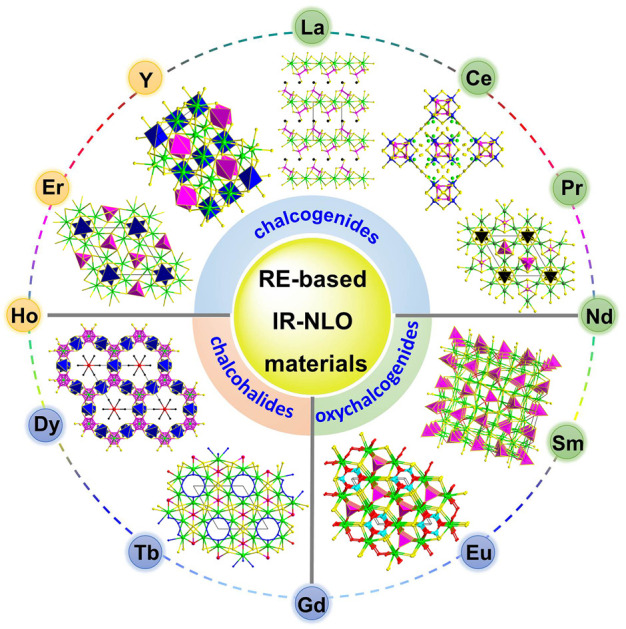

## 1. Introduction

The laser, an acronym for “Light Amplification by Stimulated Emission of Radiation,” has found extensive applications in the military, technology, medical, and industrial production sectors. The outstanding features of lasers, including excellent collimation, monochromaticity, coherence, and high brightness, make them an indispensable tool.^1–5^ However, these advantages of lasers also pose limitations to their development. The generation mechanism of lasers dictates that their wavelength is constrained. Second harmonic generation (SHG), which is a nonlinear optical (NLO) effect, enables the expansion of the working wavelengths for lasers.^6–20^ Extensive research has been carried out in the ultraviolet-visible (UV-vis) spectrum over the past few decades, with a focus on inorganic crystals known for their stability and significant effective frequency-doubling coefficient (*d*_eff_). Crystals such as KH_2_PO_4_ (KDP),^21^ KTiOPO_4_ (KTP),^22^ LiB_3_O_5_ (LBO),^23^ and β-BaB_2_O_4_ (β-BBO)^24^ have been discovered and boast excellent properties.

Due to the evolving demands of laser technology, the UV-vis spectrum is no longer sufficient. This has led to a need for research into longer and shorter wavelengths. The study of NLO crystals in the infrared (IR) and deep ultraviolet (DUV) ranges has become crucial in laser technology. Currently, the commercially available IR-NLO crystals primarily consist of AgGaS_2_,^25^ AgGaSe_2_,^26^ and ZnGeP_2_.^27^ However, they all have some unavoidable drawbacks. For instance, AgGaS_2_ and AgGaSe_2_ both have a low laser-induced damage threshold (LIDT) and AgGaSe_2_ is non-phase-matching (NPM) at 1064 nm. Additionally, ZnGeP_2_ exhibits unfavourable multi-photon absorption. Therefore, there is an urgent demand for superior commercial large crystals, which has prompted research into outstanding candidates for IR-NLO crystals. The traditional method of exploring new IR-NLO crystals involves using powder sample tests to evaluate performance instead of waiting for large-size single crystals. However, this traditional experiment is time consuming. Nowadays, new theoretical calculation techniques are often used to deepen the knowledge of the structure–performance relationship and aid in design, even using machine learning techniques such as SHG-weighted density analysis, the real-space atom-cutting technique, the dipole flexibility model, and partial response functionals.^28–38^ Through a dual analysis involving both experimental and theoretical calculations, optical testing conditions can be determined for the identification of promising candidates as ideal IR-NLO materials.

To meet the demands of commercial applications, an ideal candidate for an IR-NLO crystal must fulfil several conditions.^39–53^ Firstly, it should crystallize in a non-centrosymmetric (NCS) space group, which is a prerequisite for being a NLO crystal. Secondly, it should have a large *d*_eff_ value, preferably more than 4 pm V^−1^ (but even better if it exceeds 8 pm V^−1^), in order to enhance the conversion efficiency. Additionally, it should have a high LIDT to withstand high power densities of fundamental frequency light waves. The LIDT is positively correlated with the band gap (*E*_g_), which should be at least 3.0 eV, but it is even more desirable for it to be above 3.5 eV. Furthermore, the crystal should possess moderate birefringence (Δ*n*) falling within the range of 0.03–0.10. Excessive birefringence can lead to the walk-off effect, while a small birefringence is not conducive to achieving phase-matching (PM). In addition, the crystal should have a wide optical transparency range that encompasses two significant atmospheric windows: 3–5 μm and 8–12 μm. Finally, the perfect IR-NLO crystal should exhibit stable physical and chemical properties, maintaining stability even when exposed to the air. It should also demonstrate thermal stability, which is advantageous for growing large-sized crystals. Among the various families of materials, chalcogenides show promising potential as excellent candidates for IR-NLO crystals. Extensive research has been conducted on these materials, demonstrating their ability to achieve a superior balance between *d*_eff_ and *E*_g_ compared to other families.

Rare-earth (RE)-based chalcogenides, which are a subset of chalcogenides, continue to garner attention from researchers due to the unique properties of lanthanide elements. According to Pearson's hard and soft acid–base theory,^54^ RE^3+^ cations are categorized as hard bases. As a result, the bonds formed between these cations and anions exhibit a certain degree of ionic character, similar to alkaline earth metal cations. Additionally, the covalent nature of these bonds is primarily derived from the outer 5d and 6s orbitals. The 4f orbitals situated in the inner shell receive shielding from the outer 5s and 5p electrons, limiting their contribution to bonding. As the atomic number increases, the 4f orbitals become more dispersed, causing these electrons to not fully occupy the inner regions of the 5s and 5p orbitals. This incomplete shielding effect on the nucleus results in the phenomenon known as lanthanide contraction. Consequently, the ionic radii of Sc^3+^ and Y^3+^ are similar to those of Lu^3+^ and Er^3+^ in the lanthanide series, hence, Y is classified as a member of the RE elements.

Due to the relatively large size of RE^3+^ cations, they can accommodate higher coordination numbers. Their common coordination numbers are typically less than or equal to 9, approaching the sum of the 6s, 6p, and 5d orbitals, and can even reach up to 12. This complexity in coordination numbers contributes to the diverse geometric configurations of their polyhedra. The combination of the high coordination numbers and moderate positive charge of RE^3+^ cations enables them to effectively disperse excess negative charges on anionic groups. This dispersion reduces the mutual attraction between anionic groups, presenting an opportunity to obtain low-dimensional anionic framework materials.

So far, numerous reviews on IR-NLO materials have been published. While there are a few examples involving a small amount of RE-based materials,^55–67^ there has been no systematic overview analyzing and summarizing the fascinating RE-based chalcogenide family. In order to better illustrate the advantages of RE-based chalcogenide compounds and address the challenges in this field, this paper provides detailed explanations for 85 representative examples out of over 400 NCS compounds.^68–187^ These examples are categorized into four groups based on the various asymmetric building motifs: (1) RE-based chalcogenides containing tetrahedral motifs; (2) RE-based chalcogenides containing lone-pair-electron motifs; (3) RE-based chalcogenides containing [BS_3_] and [P_2_Q_6_] motifs; and (4) RE-based chalcohalides and oxychalcogenides. Finally, the conclusions and perspectives for RE-based chalcogenides and their derivatives are provided for further exploration.

## 2. RE-based chalcogenides containing various asymmetric building motifs

### 2.1. RE-based chalcogenides containing tetrahedral motifs

Spontaneous self-aggregation of a single [MQ_4_] anion disperses negative charges and increases the likelihood of acquiring a high-dimensional framework. RE cations exhibit excellent abilities to disperse negative charges, resulting in the formation of RE–Q bonds when charges are transferred to the anion. This creates favourable conditions for the generation of low-dimensional anionic frameworks. Additionally, the charge transfer optimizes the distribution of electrons in the compound, contributing more effectively to the NLO effects under the synergistic action of the polyhedral [REQ_*n*_] and tetrahedral [MQ_4_] asymmetric building motifs. In the La_3_LiM^IV^S_7_ (M^IV^ = Ge and Sn) system, the presence of a closed-ring structure for La–S–M^IV^ reveals the existence of d–p π bonds attributed to the La-5d orbitals and S-3p orbitals. However, the empty 5d orbitals of La^3+^ lack additional 5d electrons to form d–p back-bonding π bonds. As a result, the formation of extra bonds between RE^3+^ and Q^2−^ is hindered, limiting the enhancement of induced dipole moment oscillations. On the other hand, the lack of d electrons can be seen as an advantage as it avoids d–d and f–f transitions and enables better absorption of light. Next, the twelve systems containing tetrahedral motifs are introduced.

#### 2.1.1. RE_3_GaS_6_ (RE = Y, Dy, Ho, and Er)

**Fig. 1:**
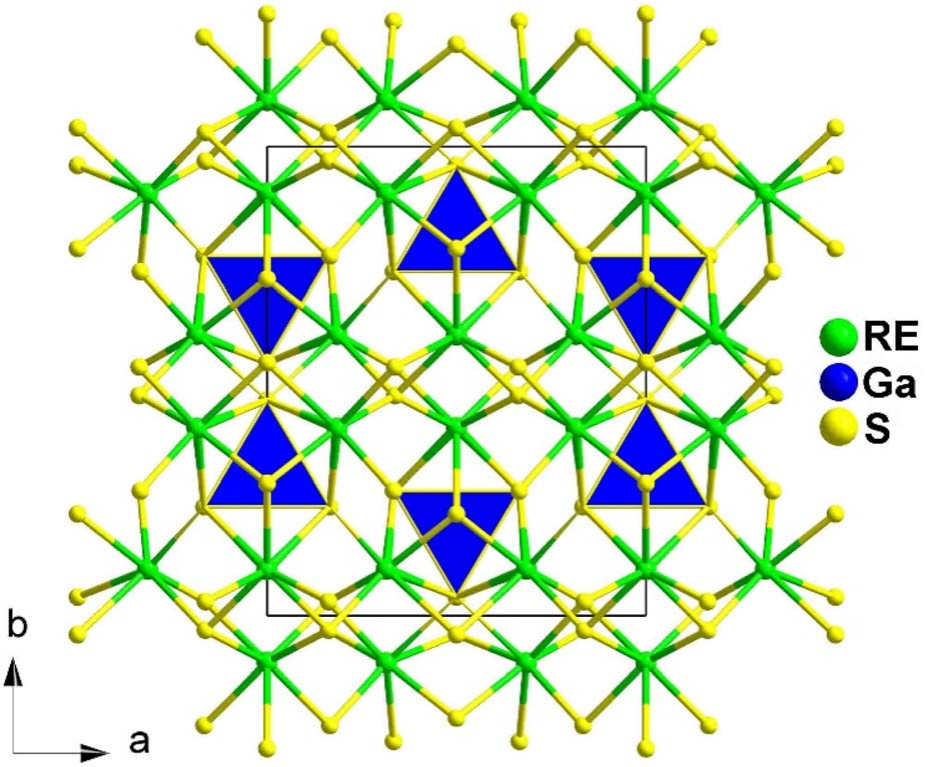
Structures of RE_3_GaS_6_ along the *c* axis with unit cell and discrete [GaS_4_] polyhedra (blue) outlined.

The crystal structure of RE_3_GaS_6_ (RE = Dy, Y, Ho, and Er) was first reported in 1971 based on powder XRD data.^68^ However, the linear and NLO properties of these compounds were not systematically studied and reported by Guo's group until 2013^69^ and 2021, respectively.^70^ High-quality single crystals of RE_3_GaS_6_ were obtained through solid-state reactions with KI as a flux. These chalcogenides are isomorphic and adopt the orthorhombic space group *Cmc*2_1_. The structures consist of two types of functional primitives: [RES_7_] monocapped trigonal prisms and [GaS_4_] tetrahedra. The overall structure can be described as [RES_7_] functional primitives constructing a 3D network, with Ga atoms occupying the tetrahedral cavities (see Fig. 1). Each tetrahedral cavity is formed by 10 [RES_7_] units, with 3 sharing an S-edge and 7 sharing an S-corner with the Ga atom.

The results of the SHG test indicate that the [RES_7_] functional primitives play a key role in the NLO activity, in addition to the [GaS_4_] tetrahedron. The *d*_eff_ of RE_3_GaS_6_ increases as the Shannon ionic radius of the RE^3+^ ion decreases, namely, in the approximate sequence Dy_3_GaS_6_ < Ho_3_GaS_6_ ≈ Y_3_GaS_6_ < Er_3_GaS_6_ when irradiated under a 2100 nm laser. This increase in *d*_eff_ can be attributed to the increasing covalent bond feature of the RE–S bonds. Upon comparing the calculation results for RE_3_GaS_6_, it can be concluded that the frontier orbitals of all compounds are primarily influenced by the RE element and S, with very little involvement of Ga. Additionally, the calculated SHG coefficients for these sulfides closely match their experimental *d*_eff_. Furthermore, the calculated value of Δ*n*, which determines the phase-matchability of IR-NLO materials, is also in line with experimental PM behaviors.

#### 2.1.2. Eu_8_Sn_4_Se_20_

As a member of the (M^II^_2_M^IV^Q_5_)_*n*_ family (M^II^ = divalent metals, Eu, Yb, Pb, and Sn; M^IV^ = group 14 metals; Q = S, Se, and Te; *n* = 1, 2, and 4), Eu_8_Sn_4_Se_20_ is the first material to demonstrate NLO activity, despite the presence of some NCS structures within this system. Although the synthesis, structure, and optical *E*_g_ of Eu_8_Sn_4_Se_20_ were reported by Dorhout and colleagues in 2001,^71^ its NLO performance was not reported until 2020 by Guo's group.^72^

**Fig. 2:**
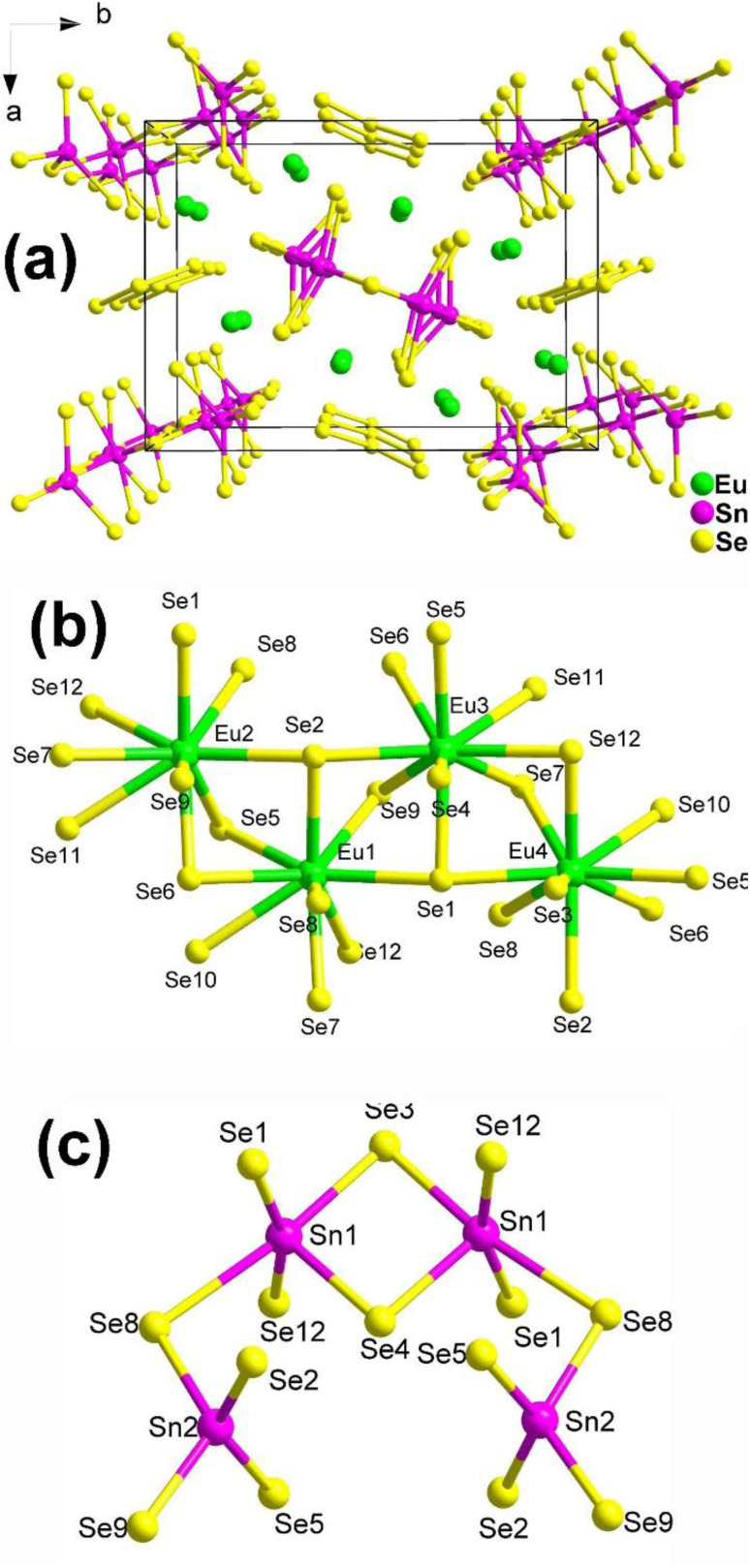
(a) Central projection of Eu_8_Sn_4_Se_20_ along the *c* axis with the unit cell outlined; (b and c) coordination environment of the crystallographically independent Eu atoms and the discrete [Sn_4_Se_14_] tetra-nuclear cluster.

The structure of Eu_8_Sn_4_Se_20_, which belongs to the orthorhombic space group *P*2_1_2_1_2, is displayed in Fig. 2a. The fundamental structural components consist of discrete [Sn_4_Se_14_] tetra-nuclear clusters and [Se_3_] trimers. The coordination environments of the Eu and Sn atoms in the structure are depicted in Fig. 2b and c. It is worth noting that Guo's group, through literature research and detailed structural analysis, pointed out in their report that the coordination geometry of Sn should be more reasonable for four-fold-coordination and six-fold-coordination, contrary to the 4- and 5-coordination mentioned in Yang's article.

Experimental results reveal that Eu_8_Sn_4_Se_20_ possesses an indirect *E*_g_ of 1.33 eV and displays antiferromagnetic-type behaviour. Most significantly, Eu_8_Sn_4_Se_20_ exhibits SHG activity (1 × α-SiO_2_ in the range of 150–210 μm under 2010 nm laser radiation), although the intensity is not particularly promising. Dipole moment measurements indicate that the majority of the dipole moment vectors of Eu/Se are in opposite directions, resulting in smaller dipole moments. This could be the primary reason for the compound's weak SHG signals.

#### 2.1.3. EuCu_2_M^IV^Q_4_ (M^IV^ = Si, Ge; Q = S, Se)

EuCu_2_GeS_4_ and EuCu_2_SnS_4_ were first synthesized in 2003 by Jaime Lanos.^73^ Furthermore, in 2009, Jennifer A. Aitken obtained EuCu_2_SnS_4_ once again.^74^ In 2019, Guo's group was able to obtain EuCu_2_GeS_4_ along with two new compounds, EuCu_2_GeSe_4_ and EuCu_2_SiS_4_, through a high-temperature solid-phase method.^75^

**Fig. 3:**
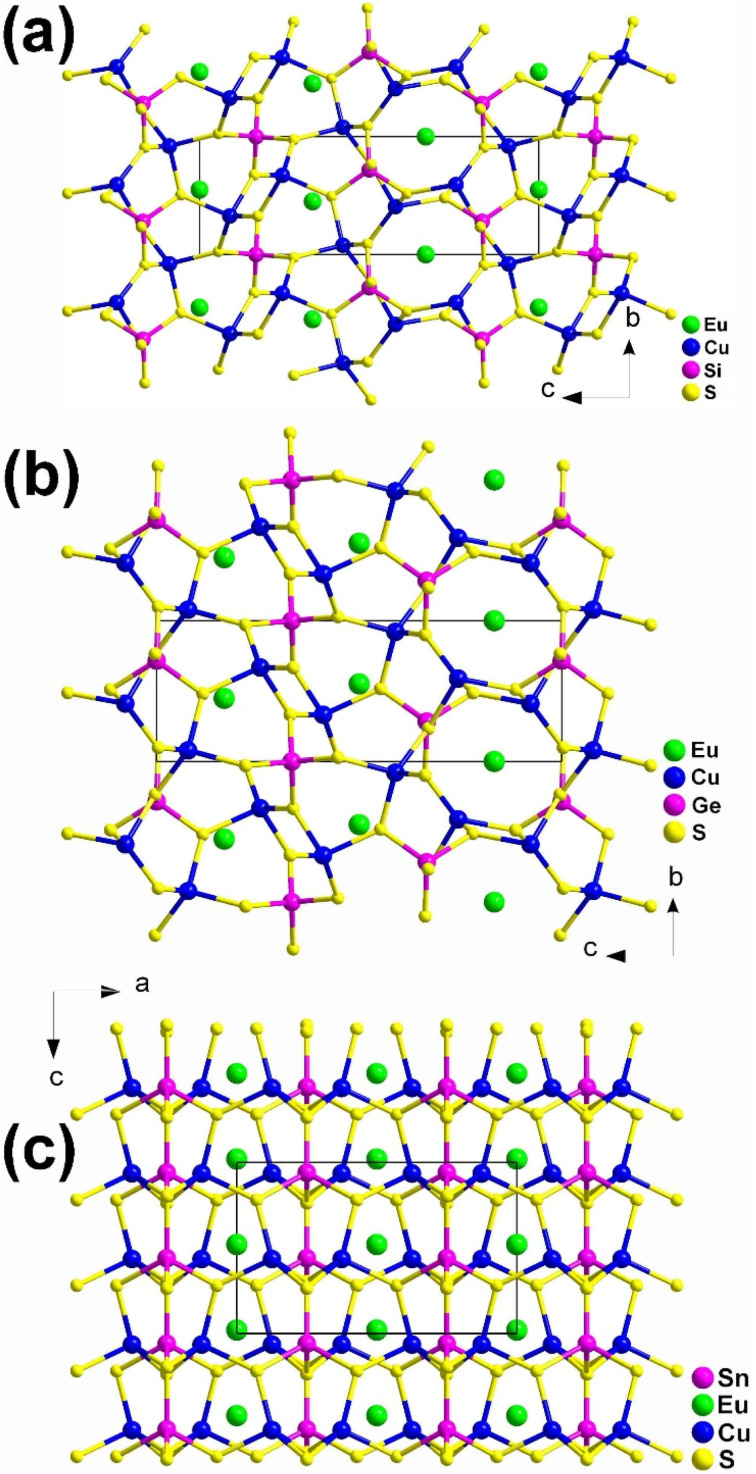
Structures of (a) EuCu_2_SiS_4_ (space group: *P*3_1_21), (b) EuCu_2_GeS_4_ (space group: *P*3_2_21), and (c) EuCu_2_SnS_4_ (space group: *Ama*2) with the unit cell outlined.

Although these compounds have the same composition ratio, they crystallize in three different spatial groups represented by the compounds EuCu_2_SiS_4_ (space group: *P*3_1_21), (b) EuCu_2_GeS_4_ (space group: *P*3_2_21), and (c) EuCu_2_SnS_4_ (space group: *Ama*2). Compounds EuCu_2_SiS_4_ and EuCu_2_GeS_4_ have similar coordination geometries and crystal structures (Fig. 3a and b), while EuCu_2_SnS_4_ has completely different ones (Fig. 3c). Similar phenomena are also common in the M^II^M^I^_2_M^IV^Q_4_ family.^188–192^ Therefore, the description of their structures will only focus on EuCu_2_SiS_4_ and EuCu_2_SnS_4_.

In EuCu_2_SiS_4_, there is 1 Eu, 1 Cu, 1 Si, and 2 S atoms in the crystallographically unique unit. This structure can be seen as a 3D network formed by [EuS_8_] bicapped trigonal prisms, with Cu and Si atoms occupying the tetrahedral cavities (Fig. 3a). Each [EuS_8_] motif links 8 neighboring [EuS_8_] motifs *via* sharing vertexes and interconnects with 2, 4, and 2 [CuS_4_] tetrahedra by sharing corners, edges, and faces, respectively.

For EuCu_2_SnS_4_, there is 1 Eu, 1 Cu, 1 Sn, and 3 S atoms in its crystallographically unique units. It also demonstrates a 3D framework made of [EuS_8_] motifs, with each [EuS_8_] motif connecting with 4 and 2 neighboring [EuS_8_] and [CuS_4_] groups by sharing corners and edges, respectively. It also links 6, 2, and 2 [SnS_4_] tetrahedra through sharing vertexes, edges, and faces, respectively (Fig. 3c).

Although EuCu_2_M^IV^Q_4_ (M^IV^ = Si, Ge; Q = S, Se) belong to the NCS space groups, no apparent SHG signals were detected under the two most commonly used wavelengths of 1064 nm and 2100 nm. This result is completely different from the previously reported isomorphic compounds of alkaline earth metal groups with strong SHG signals, and the underlying mechanism is currently unclear.

#### 2.1.4. RE_3_M_1−*x*_M′Q_7_ (RE = Y, La–Nd, Sm–Tm, Yb; M and M′ = metals or metalloids; Q = S, Se)

**Fig. 4:**
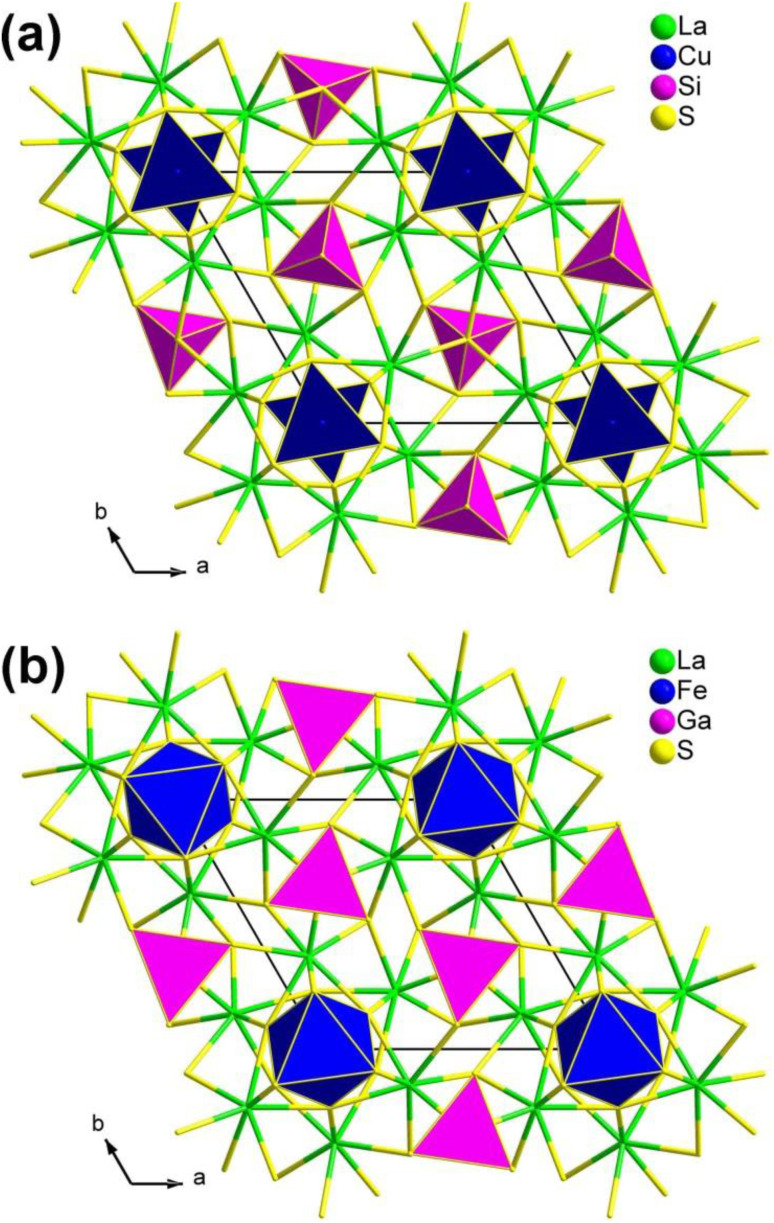
Two classic structural types in the RE_3_M_1−*x*_M^IV^Q_7_ family: (a) La_3_CuSiS_7_-type and (b) La_3_FeGaS_7_-type structures with the unit cell outlined.

The compounds RE_3_M_1−*x*_M′Q_7_, which represent a large and well-known class of chalcogenides (>400 compounds), have been extensively studied due to their intriguing structural chemistry and technologically relevant physical properties.^76–131^ From a chemical perspective, the RE_3_M_1−*x*_M′Q_7_ series can be seen as derivatives of the classic parent structure of ternary Ce_3_Al_0.67_S_7_,^193,194^ achieved through a chemical co-substitution route. In this study, two examples are presented as structural prototypes within the RE_3_M_1−*x*_M′Q_7_ family: the La_3_CuSiS_7_-type structure (Fig. 4a) and the La_3_FeGaS_7_-type structure (Fig. 4b). In these structures, the M and M′ cations are located at specific sites and are limited to certain types of cations. For example, M can be located with different metal ions with various valence states, like monovalent IA and IB elements (with 100% occupancy), bivalent IIA, IIB, and transition-metal elements (with 50% occupancy), or occasionally trivalent IIIA elements (with sectional occupancy). M′, on the other hand, is typically associated with tetravalent IVA elements. The local coordination of M, situated at the 2a Wyckoff position, is either in a trigonal [MQ_3_] or an octahedral/trigonal antiprismatic [MQ_6_] configuration, while M′, located at the 2b Wyckoff position, exhibits a tetrahedral [M′Q_4_] coordination.

It is worth mentioning that the compounds in this family exhibit significantly different SHG intensities, the underlying reasons for which have not been revealed. In 2015, Chen's research group provided an unprecedented explanation for the unique atomic distribution within this family, which is observed on both the octahedral 2*a* and tetrahedral 2*b* sites. This distribution is a direct consequence of the combined considerations of total energy and charge balance. What is even more intriguing is that, in the case of the RE_3_M_1−*x*_M′Q_7_ family, the atomic distribution predominantly determines the NLO properties. Consequently, members exhibiting a high *d*_eff_ value should adhere to the formula RE_3_M_0.5_M^IV^Q_7_.

Recently, Wu's group discovered a series of new isomorphic compounds, RE_3_LiMS_7_. Compared with the previously reported NPM behavior, these compounds exhibit useful PM features. Among them, La_3_LiGeS_7_ and La_3_LiSnS_7_ show strong *d*_eff_ values (0.7 and 1.2 × AgGaS_2_, respectively) and large LIDTs (6.0 and 2.5 × AgGaS_2_, respectively). Detailed theoretical calculations indicate that the *d*_eff_ values of the RE_3_LiMS_7_ family originate from the cooperation of intrinsic dipole moments and d–p delocalized-π-electron-induced dipole oscillations.

#### 2.1.5. EuM^II^M^IV^Q_4_ (M^II^ = Cd, Hg; M^IV^ = Ge, Sn; Q = S, Se)

It is well known that tetrahedral [MQ_4_] functional primitives, such as [M^IV^Q_4_] and [M^II^Q_4_], make a large contribution to the SHG effect. Additionally, the introduction of two or more asymmetric building units helps in further modulating the crystal structure to obtain materials with SHG activities. Based on the reported AEM^II^M^IV^Q_4_ compounds (AE = alkaline-earth metals; M^II^ = transition metals; M^IV^ = Si, Ge, and Sn, Q = S, Se), it is evident that this family is a promising candidate crystal for IR-NLO applications.^195–202^ Considering that Eu^2+^ and AE^2+^ have similarities in their ionic radii and coordination configuration in metal chalcogenides, Kang's group successfully synthesized EuCdGeQ_4_ in 2019, which was the first example containing a RE element in this family.^132^ Subsequently, they introduced Hg^2+^ with high polarizability to improve the *d*_eff_ effect in the system, successfully obtaining two Hg-based compounds, EuHgGeSe_4_ and EuHgSnS_4_.^133^

**Fig. 5:**
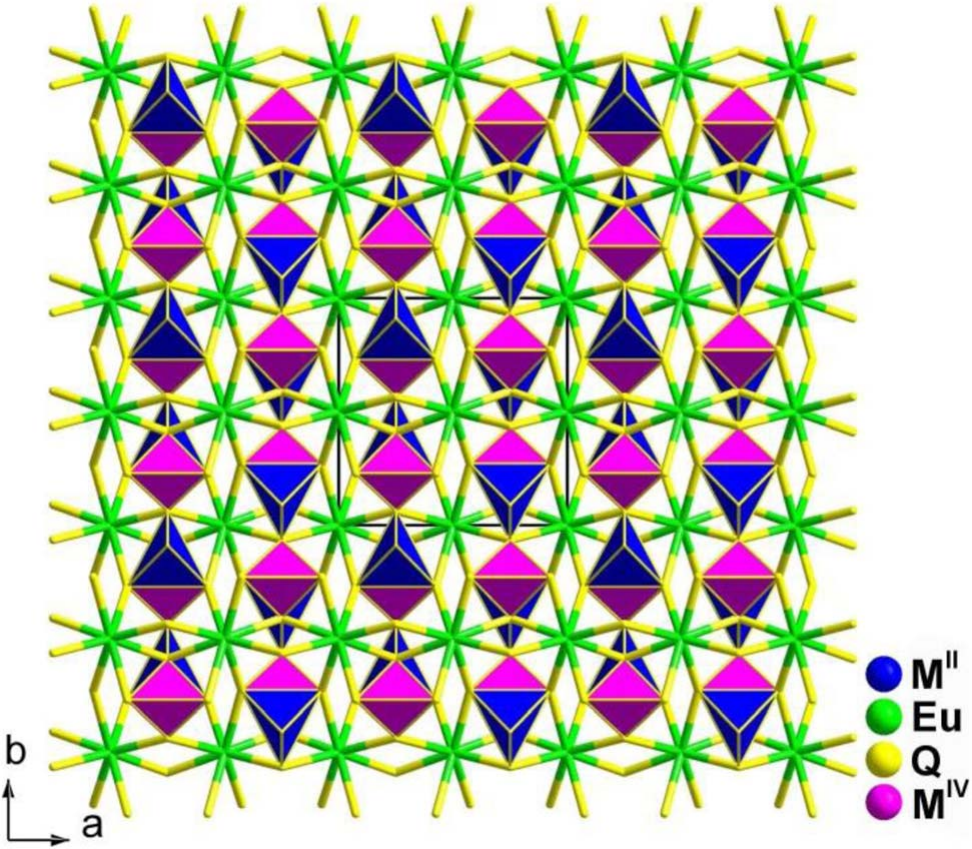
Structures of EuM^II^M^IV^Q_4_ (space group: *Ama*2) along the *c* axis with the unit cell outlined.

EuM^II^M^IV^Q_4_ (M^II^ = Cd, Hg; M^IV^ = Ge, Sn; Q = S, Se) are isostructural and crystallize in the NCS orthorhombic *Ama*2 space group. As shown in Fig. 5, a 2D covalent layer of [M^II^M^IV^Q_4_]^2−^ is made of tetrahedral [M^II^Q_4_] and [M^IV^Q_4_] motifs, and further stacks along the *a* direction with [EuQ_8_] units occupying the intervals. The geometry of the [EuQ_8_] units can be viewed as a distorted bicapped trigonal prism.

Owing to the structural traits that arrange the overall NLO-active motifs ([M_II_Q_4_] and [M^IV^Q_4_] units) in an orderly manner along a specific direction, all units exhibit strong *d*_eff_ (approximately 2–4 × AgGaS_2_@2090 nm) along with type-I PM behaviour. Furthermore, theoretical calculations and local dipole moment analyses suggest the significant contribution of distorted [M^II^Q_4_] tetrahedra to the improved *d*_eff_. These results confirm the intriguing potential applications of these materials as IR NLO crystals. This work also indicates the feasibility of replacing AE elements with RE elements in the exploration of novel IR-NLO candidates.

#### 2.1.6. La_2_Ga_2_GeS_8_ and Eu_2_Ga_2_GeS_7_

**Fig. 6:**
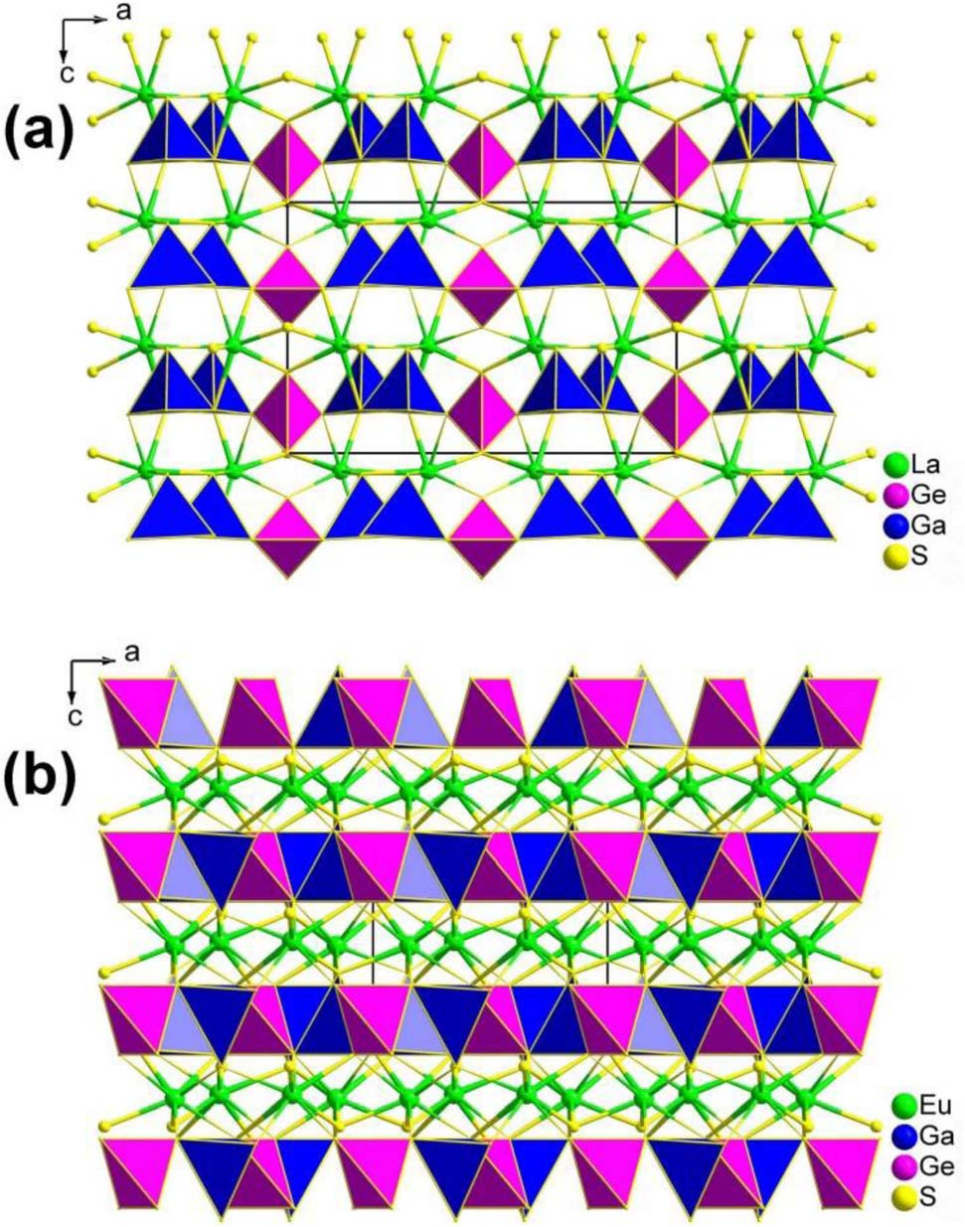
Structures of (a) La_2_Ga_2_GeS_8_ and (b) Eu_2_Ga_2_GeS_7_ along the *ac* plane with the unit cell outlined.

Two new NCS quaternary chalcogenides, La_2_Ga_2_GeS_8_ and Eu_2_Ga_2_GeS_7_, were obtained through traditional solid-state reactions in 2011 by Chen's group.^134^ La_2_Ga_2_GeS_8_ belongs to the orthorhombic space group *Cmc*2_1_, while Eu_2_Ga_2_GeS_7_ has a Ca_2_Ga_2_GeO_7_-type structure in the tetragonal space group *P*4̄2_1_*m*. They formed stacked 2D layers of interconnected [GaS_4_] and [GeS_4_] tetrahedral functional primitives with La^3+^ or Eu^2+^ cations filled into these layers (Fig. 6). However, their functional primitives are linked in different fashions. The 2D [Ga_2_GeS_8_]^6−^ layers in La_2_Ga_2_GeS_8_ are formed by wavy 1D [GaS_4_] chains that are interlinked by individual [GeS_4_] units through sharing S corners. In contrast, the 2D [Ga_2_GeS_7_]^4−^ layers in Eu_2_Ga_2_GeS_7_ are formed by [(GaS_4_)_2_] dimers connected *via* individual [GeS_4_] units.

It is worth mentioning that the number of terminal S atoms per formula plays a crucial role in determining the linkage of [GaS_4_] and [GeS_4_] functional primitives as well as the aggregation density of the anionic moieties. Theoretical analysis shows that RE cations narrow the *E*_g_ but Li does not, and that all components affect the *d*_eff_ through the electronic transitions from the S-3p state to the La/Eu/Li–S, Ga–S, and Ge–S antibonding states. This understanding may provide useful guidance for the further exploration and development of novel IR-NLO materials. Interestingly, the powder Eu_2_Ga_2_GeS_7_ exhibits a large *d*_eff_ (1.6 × AgGaS_2_@2050 nm) with type-I NPM features, a wide transparent range, and a large theoretical Δ*n*.

#### 2.1.7. Ba_2_REM^III^Q_5_ (RE = Y, Ce, Nd, Sm, Gd, Dy, and Er; M^III^ = Ga, In; Q = Se, Te)

Twenty chalcogenides with the formula Ba_2_REM^III^Q_5_ were reported in 2012 and synthesized by Wu's group using a solid-state reaction.^135,136^ They aimed to introduce f-block and p-block elements into chalcogenides to obtain unique structures and physical properties. In addition, AE metals were added to increase the *E*_g_ of the chalcogenides.

According to the report, the Ba_2_REGaSe_5_ (RE = Y, Nd, Sm, Gd, Dy, and Er) and Ba_2_REGaTe_5_ (RE = Sm and Gd) compounds are isomorphic, with a centrosymmetric triclinic system in the *P*1̄ space group. On the other hand, the Ba_2_REInSe_5_ (RE = Y, Nd, Sm, Gd, Dy, and Er), Ba_2_REGaTe_5_ (RE = Y, Dy, and Er), and Ba_2_REInTe_5_ (RE = Y, Ce, Nd, Sm, Gd, Dy, and Er) compounds belong to an NCS orthorhombic system in the *Cmc*2_1_ space group. This discussion will focus solely on the NCS compounds.

**Fig. 7:**
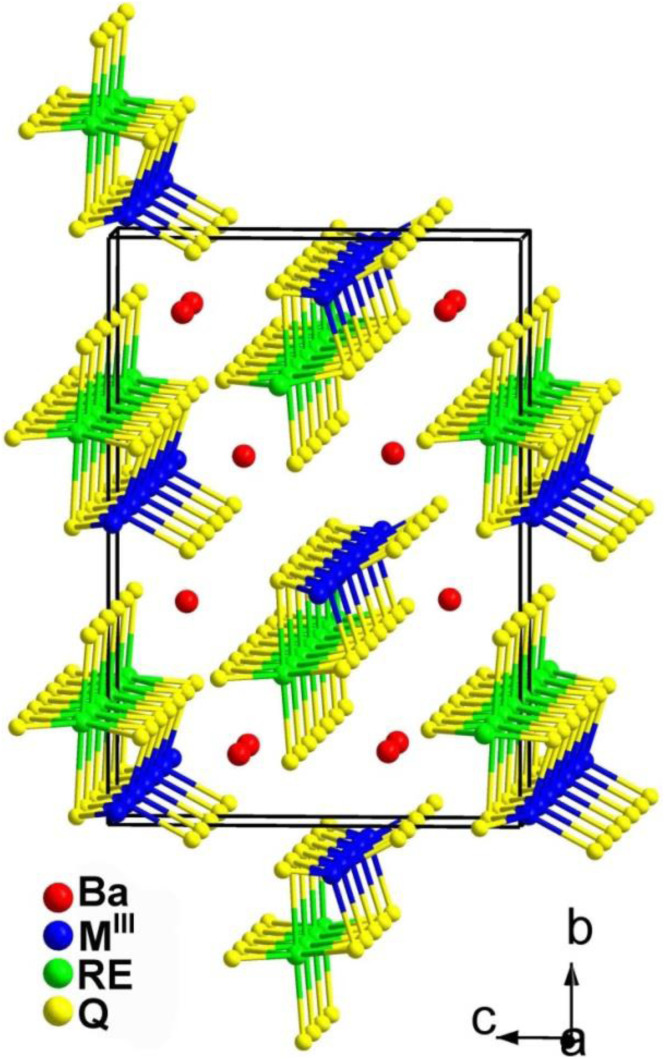
Structure of Ba_2_REM^III^Q_5_ along the *bc* plane with the unit cell outlined.

Fig. 7 shows the structure of the NCS Ba_2_REM^III^Q_5_ compounds. The structure consists of 1D [REM^III^Q_5_]^4−^ chains formed by two types of chains connected through shared Q atoms. These two types of chains are constructed by [REQ_6_] octahedra and link together through shared edges, whereas [M^III^Q_4_] tetrahedra connect through shared vertices.

Experimental results reveal that the *d*_eff_ value of Ba_2_InYSe_5_ is similar to that of AgGaSe_2_, making it the largest value among the compounds in this family. The Ba_2_REInSe_5_ (RE = Gd, Er) compounds exhibit very weak SHG responses, while the Nd, Sm, and Dy compounds show undetectable SHG responses. The detection of weak SHG signals or the failure to detect signals in the compounds of this system may be attributed to the absorption of fundamental and harmonic light by the sample. Similar phenomena have also been observed in other NCS chalcogenides.^203–205^ On the other hand, it is evident that in the Ba_2_REInSe_5_ compounds, the RE metal cation does not impact the crystal structure but does affect the intensity of the *d*_eff_.

#### 2.1.8. Ba_4_RE_2_Cd_3_S_10_ (RE = Sm, Gd, and Tb)

Ba_4_RE_2_Cd_3_S_10_ (RE = Sm, Gd, and Tb) is the only type of AE/RE/TM/Q (AE = alkaline earth elements; RE = rare-earth elements; M = main group elements; TM = d-block transition elements; and Q = chalcogen) system with an NCS space group. Previous reports on this system mainly focused on its magnetism.^206^ However, in 2022, Zhu's research group conducted a systematic investigation of its linear and NLO properties for the first time.^137^

**Fig. 8:**
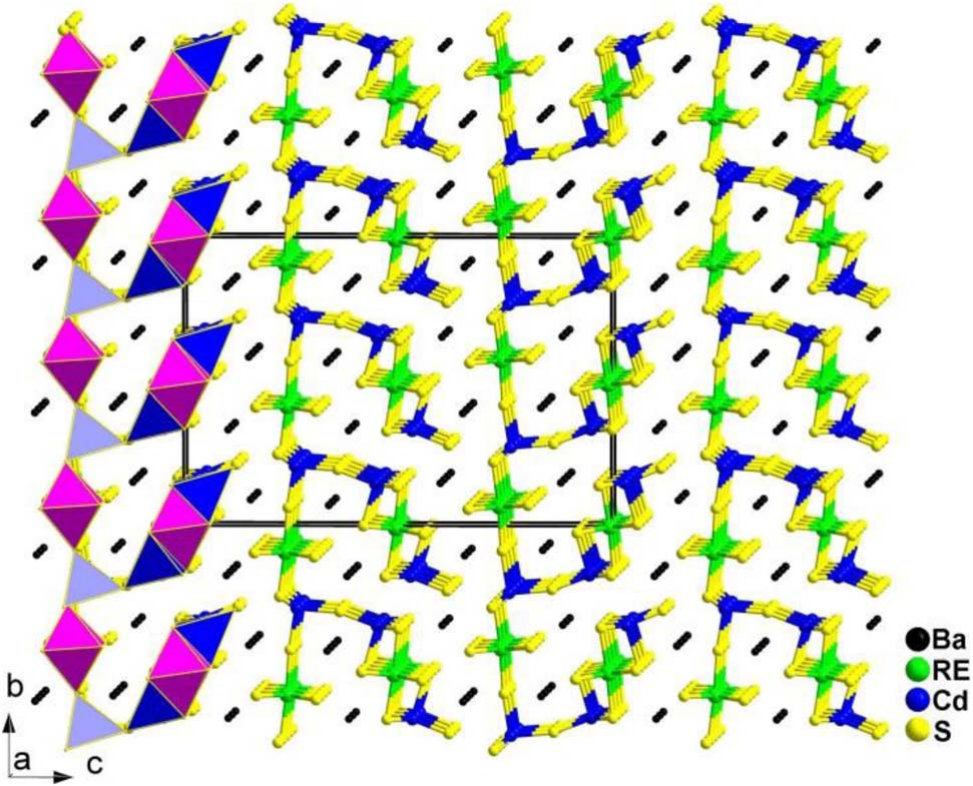
Structure of Ba_4_RE_2_Cd_3_S_10_ along the *a* axis with the unit cell outlined.

The preparation of the flat needle transparent-brown crystals of Ba_4_RE_2_Cd_3_S_10_ involved a solid-phase method of elemental mixtures in a KCl/BaCl_2_ (1 : 3) flux at 1173 K. All of them are isostructural and adopt the NCS orthorhombic space group *Cmc*2_1_ (no. 36). Fig. 8 displays a view of the structure of Ba_4_RE_2_Cd_3_S_10_ on the *bc* plane, where the most prominent feature is the 2D complex anionic [RE_2_Cd_3_S_10_]^8−^ layers stacked in an *ABABAB* fashion along the *ab*-plane, with discrete Ba^2+^ cations located in between.

Remarkably, Ba_4_Sm_2_Cd_3_S_10_ exhibits a strong *d*_eff_ (1.8 × AgGaS_2_) and a significantly higher LIDT (14.3 × AgGaS_2_). This is the first case of a quaternary AE/RE/TM/Q system possessing an IR-NLO property. Furthermore, the theoretical results for the structure–activity relationships suggest that the combined action of different types of NLO-active motifs (*i.e.*, [RES_6_] and [CdS_4_]) contributes to the SHG activity.

#### 2.1.9. LaAEM^III^_3_S_7_ (AE = Sr, Ca; M^III^ = Al, Ga)

LaCaGa_3_S_7_ was first synthesized in 1987, and only its structure was reported at that time.^138^ In 2022, Zhang's team synthesized LaAEGa_3_S_7_ (AE = Ca, Sr) again in a study that compared their specific properties and revealed a series of changes in analogues involving oxides, sulfides, and oxysulfides.^139^ Subsequently, they obtained two other isomorphic LaAEAl_3_S_7_ (AE = Ca, Sr) compounds through homologous substitution.^140^

**Fig. 9:**
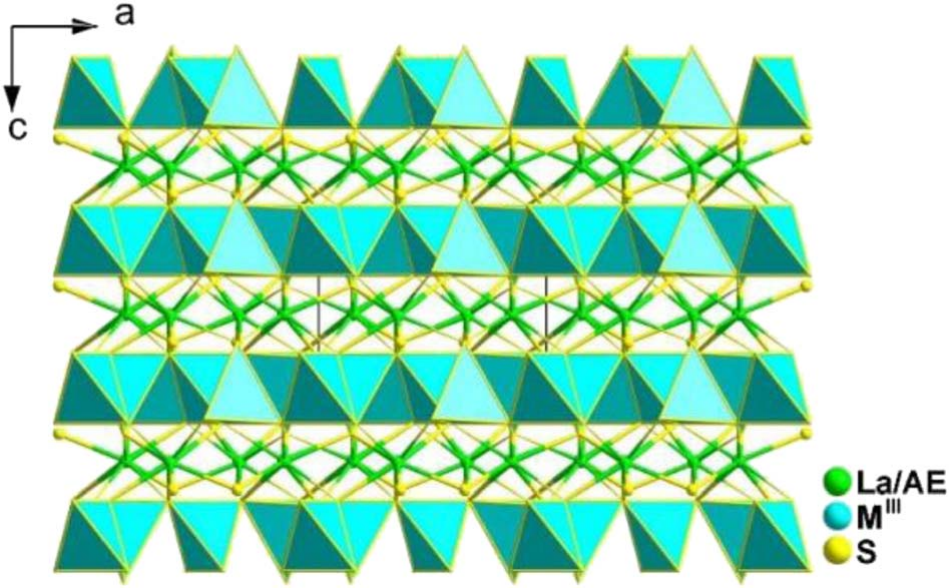
Structure of LaAEM^III^_3_S_7_ along the *b* axis with the unit cell outlined.

LaAEM^III^_3_S_7_ adopt the *P*4̄2_1_*m* space group of the tetragonal system. In the structure, La and AE atoms co-occupy the same position with occupation ratios of 50% and 50%. As shown in Fig. 9, one [M^III^S_4_] motif is connected with four other [M^III^S_4_] motifs to generate a [M^III^_5_S_16_]^17−^ windmill cluster and further interlink together to form a 2D Cairo pentagonal layer along the *c* direction. Polyhedral [(La/AE)S_8_] groups filled in the interlayers to bridge neighbouring layers together to build the overall 3D framework.

It should be noted that the 2D Cairo pentagonal layer in LaAEAl_3_S_7_ contributes to their excellent optical performance. This includes a large *d*_eff_ (0.8–1.1 times that of AgGaS_2_), a broad optical *E*_g_ of 3.76–3.78 eV (the first known cases to achieve the breakthrough of the “3.5 eV wall” among all RE-based IR-NLO chalcogenides), high LIDTs (approximately 9 times that of AgGaS_2_), and moderate Δ*n* (0.059 for the Ca-compound and 0.077 for the Sr compound at 2000 nm) for PM. These findings offer a new structural guidance route for the development of high-performance IR-NLO chalcogenides.

#### 2.1.10. RE_2_AE_3_M^IV^_3_S_12_ (RE = La, Pr, Nd, Sm, Gd, Ho, and Er; AE = Sr, Ca; M^IV^ = Si, Ge, and Sn)

Currently, a significant challenge in the field of IR-NLO is how to effectively combine and select asymmetric functional primitives to achieve NCS structures and strong *d*_eff_. In 2023, Wu and co-workers achieved successful synthesis of 29 compounds by combining flexible [SnS_*n*_] (*n* = 5 and 6) functional primitives with highly positively charge-balanced ions, such as RE^3+^ and AE^2+^ cations. These compounds included 12 NCS thiostannates, RE_2_Sr_3_SnS_12_ (space group: *Pmc*2_1_), and RE_2_Ca_3_Sn_3_S_12_ (space group: *P*6̄2*m*), as well as 17 CS compounds, RE_2_AE_3_Ge_3_S_12_ and RE_2_AE_3_Si_3_S_12_, constructed from rigid [GeS_4_] or [SiS_4_] tetrahedra.^141,142^

**Fig. 10:**
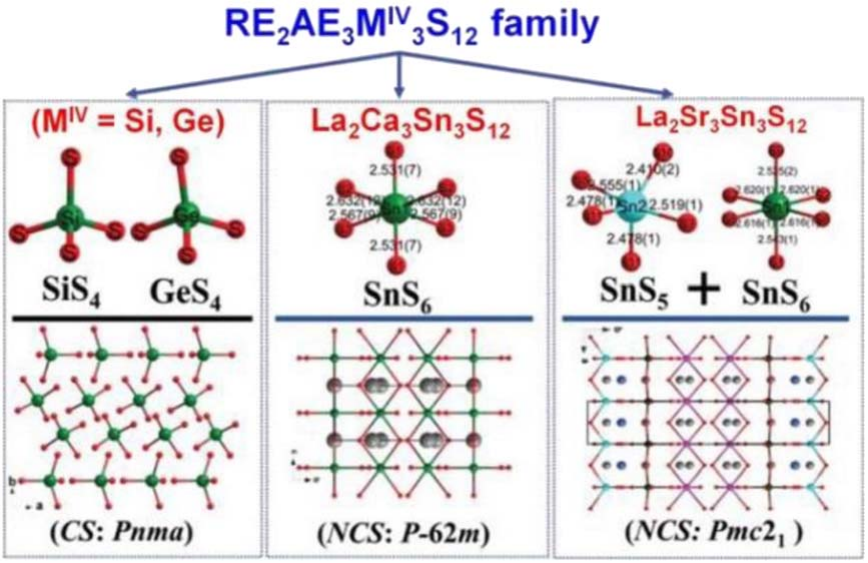
CS-to-NCS structural transformation with varying anionic motifs in the RE_2_AE_3_M^IV^_3_S_12_ family. This figure has been adapted from ref. 142 with permission from Wiley-VCH, copyright 2024.

La_2_Sr_3_M^IV^_3_S_12_ (M^IV^ = Si, Ge), La_2_Ca_3_Sn_3_S_12_, and La_2_Sr_3_Sn_3_S_12_ serve as three typical examples to illustrate the inherent structural features of the family (Fig. 10). The structure of La_2_Sr_3_M^IV^_3_S_12_ (M^IV^ = Si, Ge) exhibits isolated [M^IV^S_4_] groups, and all the isolated [M^IV^S_4_] groups located in the *c* direction are symmetrical. Additionally, the [LaS_8_] polyhedra are connected together to generate a 3D framework, while the polyhedral [SrS_7_] and [SrS_9_] groups are interconnected to create an isolated triple-chain structure. In the La_2_Ca_3_Sn_3_S_12_ structure, La and Ca atoms occupy the same positions and Sn atoms are coordinated to 6 S atoms to build the [SnS_6_] octahedron. The [SnS_6_] units interconnect with each other to form edge-sharing 1D [(SnS_4_)]_*n*_ chains, which are situated within the tunnels encircled by the polyhedral [(La/Ca)S_7_] and [(La/Ca)S_9_] motifs. It is worth noting that [Sn^IV^S_5_] and [Sn^IV^S_6_] units were only found in the structures of La_2_Sr_3_Sn_3_S_12_. The [SnS_5_] and [SnS_6_] units have no connection, and each forms isolated 1D chains with various linking modes. For instance, [SnS_5_] units are interlinked together *via* sharing vertexes to build 1D [(SnS_4_)]_*n*_ chains, while [SnS_6_] units interconnect with each other to generate edge-sharing 1D [(SnS_4_)]_*n*_ chains. The arrangement orientation of [SnS_5_] units in the unit cell demonstrates a nearly uniform arrangement along the *ab* plane.

The remarkable CS-to-NCS structural transition (from *Pnma* to *P*6̄2*m* to *Pmc*2_1_) observed in the RE_2_AE_3_M^IV^_3_S_12_ family indicates that the substitution of tetrahedral [GeS_4_]/[SiS_4_] motifs with [SnS_*n*_] disrupts the original symmetry, resulting in the formation of the necessary NCS structures.

Significantly, the [SnS_5_] unit exhibits strong polarization anisotropy and hyperpolarizability, leading to significant performance improvements in NPM *d*_eff_ for compounds like La_2_Ca_3_Sn_3_S_12_ (1.4 × AgGaS_2_ and Δ*n* = 0.008), compared to the strong PM *d*_eff_ observed in compounds like La_2_Sr_3_Sn_3_S_12_ (3.0 × AgGaS_2_ and Δ*n* = 0.086). These findings demonstrate [SnS_5_] to be a promising “NLO-active motif”.

By incorporating flexible [SnS_*n*_] (*n* = 5 and 6) NLO-active motifs into the crystal structure, this study offers a viable approach for designing novel NCS thiostannates that exhibit very large SHG effects and significant optical anisotropy.

#### 2.1.11. AREM^IV^Q_4_ (A = Li, K, Rb, and Cs; RE = Y, La–Nd, Sm–Yb; M^IV^ = Si, Ge; Q = S, Se)

Systematic exploratory efforts have resulted in the discovery of 46 new chalcogenides in this system. These include the LiRESiS_4_ (RE = La, Ce), KREM^IV^Q_4_ (RE = Y, La–Nd, Eu–Tb; M^IV^ = Si, Ge; Q = S, Se), and AREM^IV^Q_4_ (RE = La–Nd, Eu–Yb; M^IV^ = Si, Ge; Q = S, Se).^143–148^ Although they share the same 1 : 1 : 1 : 4 stoichiometry, these compounds belong to different crystal systems and exhibit different structural symmetries.

**Fig. 11:**
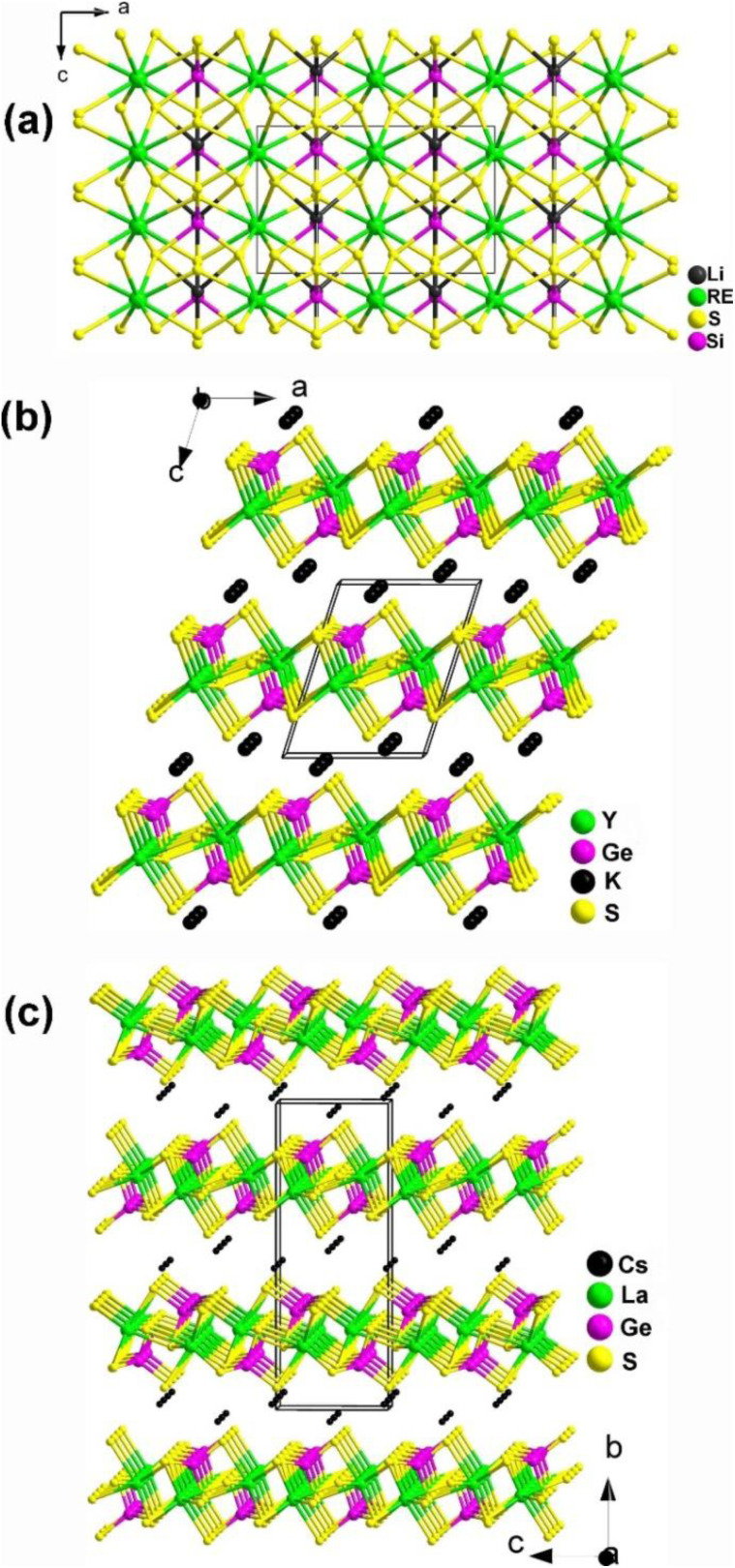
Structures of (a) LiRESiS_4_ (space group: *Ama*2), (b) KYGeS_4_ (space group: *P*2_1_), and (c) CsLaGeS_4_ (space group: *P*2_1_2_1_2_1_) with the unit cell outlined.

Two new RE-based thiosilicates, specifically, RELiSiS_4_ (Ln = La and Ce), were successfully discovered by aliovalent substitution based on SrCdSiS_4_ as a parent compound in 2023 by Mao's group. They were synthesized using Li_2_S, RE_2_S_3_, SiS_2_, and S in a molar ratio of 3 : 1 : 3 : 8 through a high-temperature solid-state method at 1073 K. They are isostructural and belong to the orthorhombic space group *Ama*2 (no. 40). As shown in Fig. 11a, a 2D covalent layer of [LiSiS_4_]^3−^ is constructed by tetrahedral [LiS_4_] and [SiS_4_] motifs, which further stacks along the *a* direction with [RES_8_] units occupying the intervals. Interestingly, this is the first observation of this linking mode in the Li–Si–S system. RELiSiS_4_ (RE = La and Ce) also exhibit perfectly balanced properties for IR-NLO chalcogenides: the largest *d*_eff_ among all reported thiosilicates (2.0 and 2.1 × AgGaS_2_@2050 nm for LaLiSiS_4_ and CeLiSiS_4_, respectively) with PM ability, high LIDTs (14 and 9 × AgGaS_2_@1064 nm), large *E*_g_ (3.71 and 2.92 eV), wide IR transmission window (0.35–18 μm), and good thermal stability above 973 K. This study not only enriches the structural chemistry of RE-based thiosilicates, but also offers an efficient method to design novel high-performance IR-NLO candidates.

KREM^IV^Q_4_ compounds (RE = Y, La–Nd, Eu–Tb; M^IV^ = Si, Ge; Q = S, Se) belong to the NCS monoclinic space group *P*2_1_ (no. 4) and are isostructural. Therefore, for the purposes of this description, only the structure of KYGeS_4_ will be detailed as a representative. This compound was successfully synthesized in 2021 by Mei's group using a charge transfer engineering strategy. Fig. 11b shows that the [YS_7_] and [GeS_4_] motifs are interconnected by sharing polyhedral edges, and the [YS_7_] units are linked to one another through sharing vertexes to generate the infinite 2D [YGeS_4_]_*n*_ layers. The interlayer interstices are filled by K^+^ cations to maintain charge balance. It is noteworthy that both [GeS_4_] and [YS_7_] polyhedra are uniformly arranged along the *ab* plane, which facilitates the positive superposition of microscopic second-order susceptibility, resulting in a strong *d*_eff_. KYGeS_4_ overcomes the *E*_g_ limitation found in rare earth chalcogenides with the largest *E*_g_ (3.15 eV) within this material system, while displaying a significant *d*_eff_ (*ca.* 1.0 × AgGaS_2_). Moreover, KYGeS_4_ is the first RE-based chalcogenide to surpass the “3.0 eV wall” observed in IR-NLO crystals.

Isostructural AREM^IV^Q_4_ compounds (RE = La–Nd, Eu–Yb; M^IV^ = Si, Ge; Q = S, Se) were synthesized by Loye and co-workers using a molten alkali halide flux growth method in 2019. All of them adopt the NCS monoclinic space group *P*2_1_2_1_2_1_ (no. 19), and in this discussion, we will focus on the structure of CsLaGeS_4_ as an example. CsLaGeS_4_ can be easily obtained by adding Ge to the reaction mixture of La_2_S_3_ and S in the CsCl/KCl eutectic flux. In the structure of CsLaGeS_4_, there are [LaS_7_] monocapped trigonal prisms and [GeS_4_] tetrahedrons. These polyhedral [LaS_7_] motifs and tetrahedral [GeS_4_] units interlink together through the sharing edge to generate the infinite 2D [LaGeS_4_]^−^ slabs in the *ac* plane. The Cs^+^ cations fill in the gaps between the layers and act as charge balancers. Unfortunately, only CsLaGeS_4_ was found to be SHG-active, exhibiting nearly half the intensity of α-SiO_2_ when irradiated with a Nd:YAG 1064 nm laser. This compound also behaves as a semiconductor with an *E*_g_ of 3.60 eV based upon UV-vis diffuse reflectance measurements.

#### 2.1.12. K_3_REP_2_S_8_ (RE = Y, Ho, and Er)

**Fig. 12:**
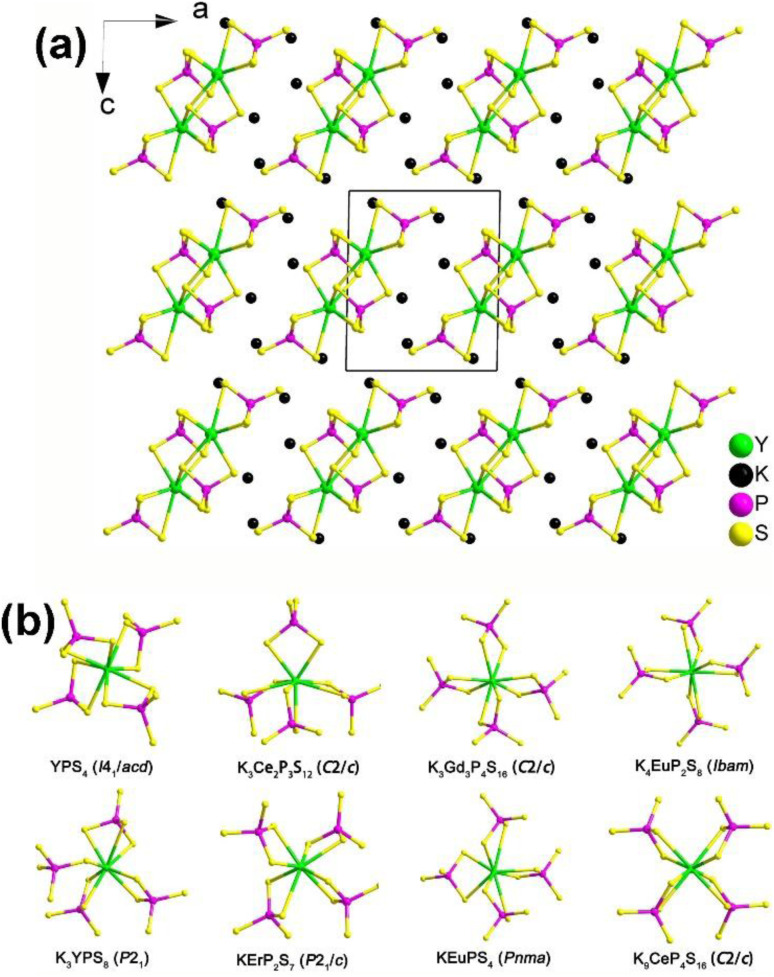
(a) Structure of K_3_YP_2_S_8_ along the *ac* plane with the unit cell outlined and (b) comparison on the local coordination modes between [RES_8_] and [PS_4_] motifs in the reported K/RE/P(V)/S family.

In 2023, a new series of RE-based thiophosphates, K_3_REP_2_S_8_, was successfully prepared and systematically investigated by Wu and co-workers.^149^ Microcrystals of K_3_REP_2_S_8_ were obtained with the stoichiometric K, RE_2_S_3_, P, and S by spontaneous crystallization. Single-crystal XRD structural refinement indicates that K_3_REP_2_S_8_ adopts two different types of space groups: NCS *P*2_1_ (RE = Y, Ho, and Er) and CS *P*2_1_/*c* (RE = Pr, Sm, and Gd). The similarities and differences in their structures are as follows: (i) they possess the same asymmetric motifs consisting of 3 K, 1 RE, 2 P, and 8 S sites but different *Z* values (*Z* = 2 for NCS; *Z* = 4 for CS) in a unit cell; (ii) [RES_8_] dodecahedra connect each other *via* sharing vertexes to generate the 1D isolated [RES_7_]_*n*_ chains (Fig. 12a) in NCS structure, which is different than the 1D [RES_6_]_*n*_ chain formed by edge-sharing [RES_8_] units in the CS structure; (iii) isolated [PS_4_] motifs link with [RES_8_] units to produce the 1D [REP_2_S_8_]^3−^ chains through sharing faces, edges, and vertexes in the NCS structure, which is distinguished from the interconnections (only edge-sharing) between the [RES_8_] and [PS_4_] motifs in the CS structure. In addition, the inherent connection mode between the [RES_8_] and [PS_4_] motifs in the K–RE–P^V^–S system was investigated (Fig. 12b) to evaluate the relationships between local asymmetry and the centrality of the overall network. The survey results indicate that most of them belong to the CS space group, and due to local symmetry, have edge sharing patterns between [RES_8_] and [PS_4_] motifs. That is, the CS-to-NCS structural transformation in the K_3_REP_2_S_8_ family can be attributed to the RE cation-size effect.

Remarkably, NCS K_3_REP_2_S_8_ displaying strong PM *d*_eff_ (1.1–1.4 × AgGaS_2_) and large optical anisotropy (Δ*n* = 0.084–0.099) were also proven to be promising IR-NLO candidates. The SHG density and dipole moment calculations indicate that the synergistic effect between the [RES_8_] and [PS_4_] units creates an inherent NLO origin, which verifies that RE-based thiophosphates can be regarded as an excellent research system for exploring novel IR-NLO chalcogenides.

### 2.2. RE-based chalcogenides containing lone-pair-electron motifs

The unique structural characteristics of polyhedra formed by cations that contain a stereochemically active lone pair (SCALP) give rise to a strong local built-in electric field, resulting in a significant dipole moment. As a result, neighbouring polyhedra with a SCALP often connect in opposite orientations, increasing the likelihood of crystallizing in an NCS space group.^207–214^ The inclusion of highly positively charged RE cations effectively creates a built-in electric field within their immediate surroundings. This takes advantage of the electrostatic interaction between the SCALP electrons and the RE cations to align the initially different orientations, thereby facilitating the macroscopic overlap of the static dipole moments. This alignment enhances the chances of crystallization in an NCS space group. Three types of RE-based chalcogenides containing lone-pair-electron motifs are presented below: RE_8_Sb_2_S_15_ (RE = La, Ce, Pr, and Nd), La_2_CuSbS_5_, and RE_4_M^III^SbQ_9_ (RE = Y, La, Pr, Nd, Sm, Tb, Dy, and Ho; M^III^ = Ga, In; Q = S, Se).

#### 2.2.1. RE _8_Sb_2_S_15_ (RE = La, Ce, Pr, and Nd)

**Fig. 13:**
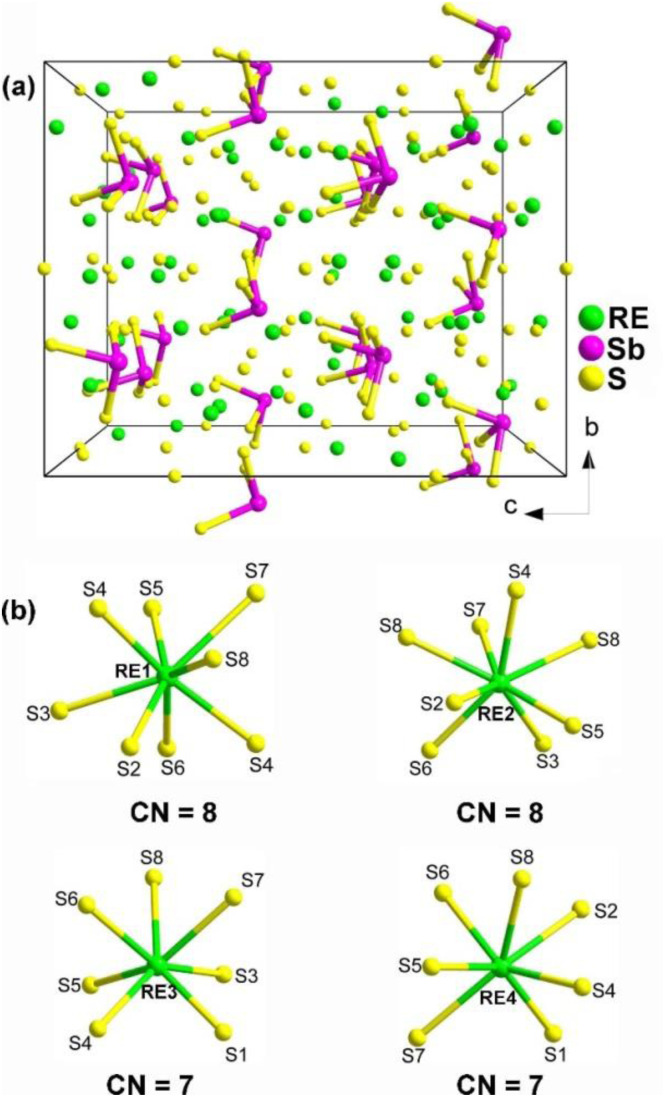
(a) Central projection of RE_8_Sb_2_S_15_ with the unit cell outlined and (b) coordination environment of crystallographically independent RE atoms.

Through the combination of Sb with stereochemically active lone-pair electrons and RE with high coordination numbers (CN), Zhao and co-workers successfully synthesized RE_8_Sb_2_S_15_ (RE = La, Pr, and Nd) in 2015.^150^ However, Nd_8_Sb_2_S_15_ could not be obtained as a single phase.^151^ Although Pr_8_Sb_2_S_15_ had previously been obtained in 1981, its SHG performance was not reported.^152^ In 2017, Zhao obtained Ce_8_Sb_2_S_15_*via* high-temperature solid-state synthesis.^153^ The lanthanide contraction from La to Nd does not affect the crystal structure of these compounds, as they are isostructural and adopt the NCS tetragonal *I*4_1_*cd* space group. The structure of RE_8_Sb_2_S_15_ with the unit cell outlined is shown in Fig. 13a. The structure consists of discrete [SbS_3_]^3−^ trigonal pyramids arranged approximately along the [001] direction, filled by RE^3+^ cations and S^2−^ anions. RE_1_ and RE_2_ have normal monocapped trigonal prismatic coordination environments, whereas the other two crystallographically unique RE positions, RE3 and RE4, are occupied in approximately square antiprismatic modes (Fig. 13b). Therefore, the formula of RE_8_Sb_2_S_15_ can be written as [(RE^3+^)_8_([SbS_3_]^3−^)_2_(S^2−^)_9_].

The compound La_8_Sb_2_S_15_ shows an *E*_g_ of 2.30 eV and a high effective *d*_eff_ of 1.2 × AgGaS_2_ (at 74–106 μm under 2050 nm laser irradiation) with NPM behavior. However, no significant *d*_eff_ was observed for Pr_8_Sb_2_S_15_, which may be linked to its poor crystallinity.

#### 2.2.2. La_2_CuSbS_5_

A millimeter-grade single crystal of La_2_CuSbS_5_ was first prepared by Zhu's group in 2019 using a BaCl_2_/CsBr flux at a temperature of 1273 K.^154^ Although this compound was discovered in 2016, no properties were reported at that time due to the inability to obtain a pure phase.^155^

**Fig. 14:**
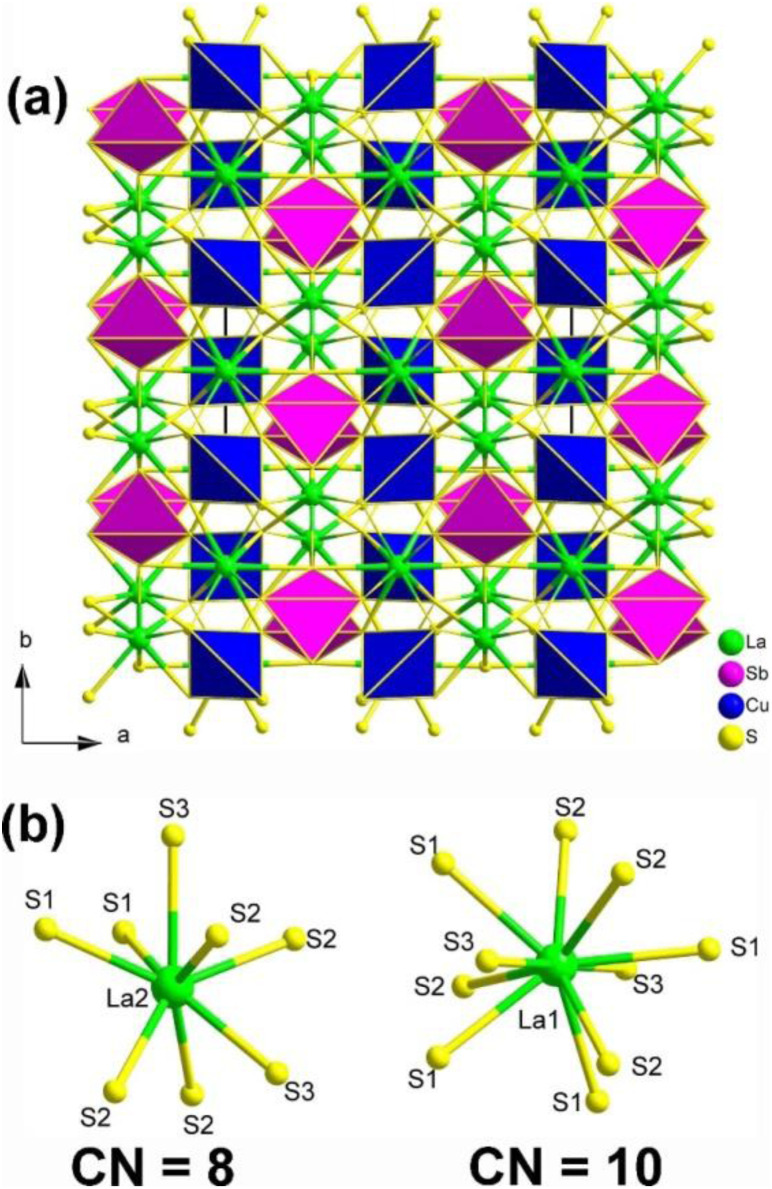
(a) Structure of La_2_CuSbS_5_ along the *ab* plane with the unit cell outlined; (b) coordination environment of crystallographically independent La atoms.

Single-crystal XRD result indicated that La_2_CuSbS_5_ belongs to the NCS orthorhombic system with the space group *Ima*2 (no. 46). The structure of La_2_CuSbS_5_ made of several distinct units, namely, [LaS_10_] polyhedra, [CuS_4_] tetrahedra, and [SbS_4_] pyramids. Fig. 14a depicts the projection of La_2_CuSbS_5_ viewed down the *ab* plane. The 3D network is constructed from two types of 2D layers (La/Cu/S and La/Sb/S) that are interconnected through shared S–S apexes and edges. The detailed coordination environment of the La atoms is shown in Fig. 14b.

Moreover, La_2_CuSbS_5_ can be seen as a result of the stereochemically active lone pair induction (SCALPI) strategy, which is based on the previously known CS La_2_CuInS_5_. Investigation into its optical properties showed that La_2_CuSbS_5_ displays a suitable PM *d*_eff_ (0.5 × AgGaS_2_) and a large LIDT (6.7 × AgGaS_2_), making it a promising candidate for IR-NLO materials. Furthermore, theoretical calculations support the notion that the *d*_eff_ can be attributed to the synergistic effects of the functional primitives, with a particular emphasis on the [SbS_4_] motifs. These results confirm the practicality of utilizing the SCALPI strategy for the design and exploration of new IR NLO crystals.

#### 2.2.3. RE_4_M^III^SbQ_9_ (RE = Y, La, Pr, Nd, Sm, Tb, Dy, and Ho; M^III^ = Ga, In; Q = S, Se)

**Fig. 15:**
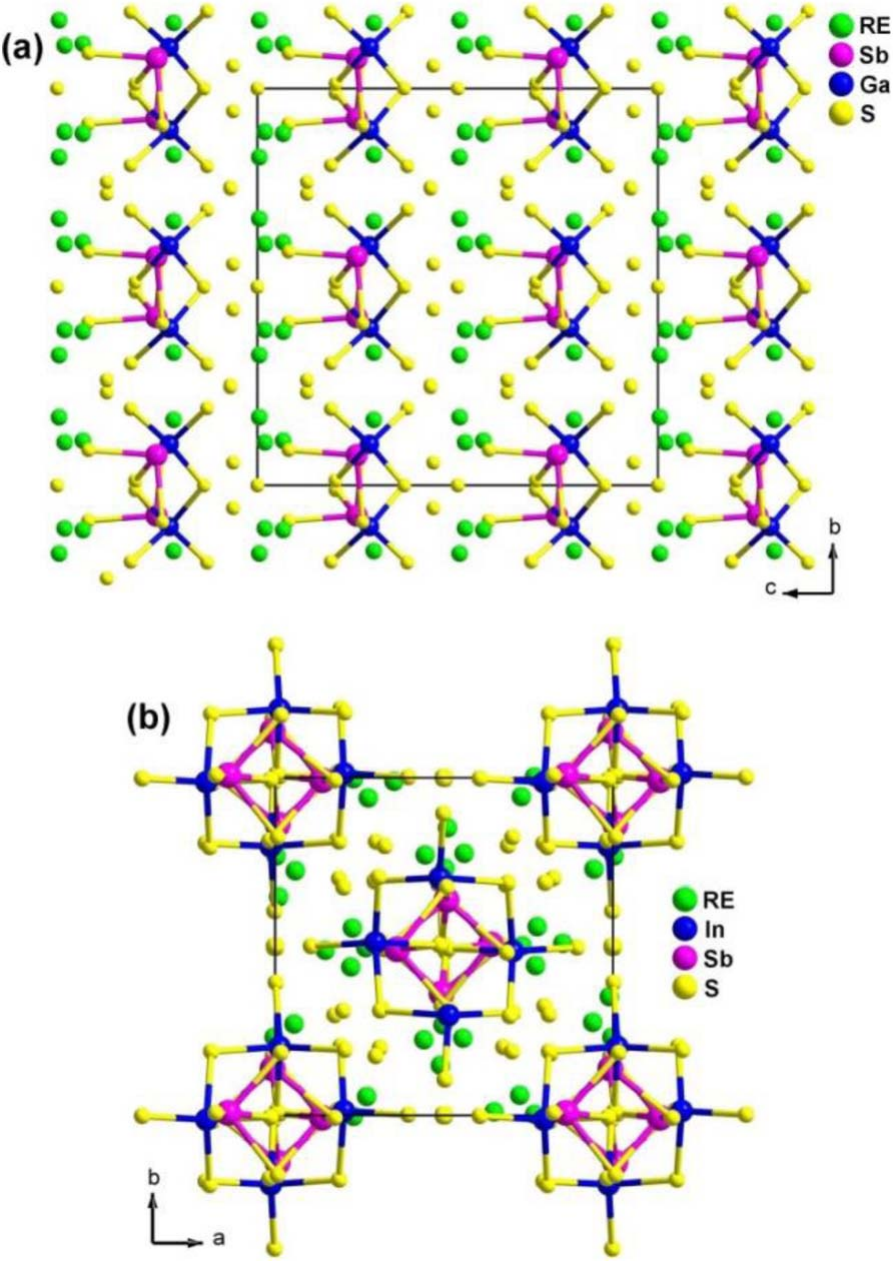
Structures of (a) RE_4_GaSbS_9_ and (b) RE_4_InSbS_9_ with the unit cell outlined.

Single-crystal XRD structural refinement indicates that RE_4_M^III^SbQ_9_ adopts two different types of crystal structures: RE_4_GaSbS_9_ (RE = Pr, Nd, Sm, Gd to Ho and Y) and RE_4_InSbQ_9_ (RE = La to Nd, Sm, Y; Q = S, Se).^156–162^ As shown in Fig. 15, the structures of these compounds have both similarities and differences. Firstly, the isostructural Ga-analogues belong to the orthogonal system [space group: *Aba*2 (no. 41)], while the isostructural In-analogues crystallize in the tetragonal system [space group: *P*4_1_2_1_2 (no. 92) or *P*4_3_2_1_2 (no. 96)]. Secondly, they have similar asymmetric motifs consisting of 4 RE, 1 M^III^, 1 Sb, and 9 Q sites, as well as the same *Z* values in a unit cell. Lastly, in the RE_4_GaSbS_9_ compounds, the [Ga_2_S_6_] and [Sb_2_S_5_] functional primitives are arranged around a 2-fold screw axis, with neighboring dimers oriented in the same direction (Fig. 15a). In contrast, in the RE_4_GaSbQ_9_ compounds, the [In_2_Q_6_] and [Sb_2_Q_5_] functional primitives are arranged in the opposite direction (Fig. 15b).

The experimental results demonstrate that RE_4_GaSbS_9_ and RE_4_InSbQ_9_ exhibit different NLO performances. The former is NPM while the latter is PM under the same test conditions. For example, among the Ga analogues, Sm_4_GaSbS_9_ shows the strongest *d*_eff_ (3.8 × AgGaS_2_ in the particle size of 46–74 μm) with NPM features at 2050 nm. Among the In analogues, La_4_InSbS_9_ exhibits a strong *d*_eff_ (1.5 × AgGaS_2_ in the particle size of 150–210 μm) with PM features at 2050 nm.

The theoretical results suggest that the SHG response of RE_4_GaSbS_9_ can be attributed to the electronic transitions from the S-3p states in the valence bands to the Sb–S and RE–S antibonding states. On the other hand, the SHG response of RE_4_InSbQ_9_ is attributed to the thermal vibrations of the lattice.

### 2.3. RE-based chalcogenides containing [BS_3_] and [P_2_Q_6_] motifs

The [BS_3_] motif, with its distinctive planar conjugated π_4_^6^ electron structure, is a crucial functional unit. Its introduction not only benefits the synergistic enhancement of NLO response and LIDT, but also has a significant impact on birefringence. By anchoring RE cations to the [BS_3_] unit, the arrangement of the [BS_3_] unit is optimized. The bonding interaction between them synergistically enhances the contribution to conductive bands, reinforcing the contribution to the empty virtual–hole transition process by the low-lying 4f or 5d orbitals in the rare earth ions and the π* anti-bonding orbitals of the [BS_3_] unit. For the crossed ethane configuration of the [P_2_Q_6_] unit, the 6 exposed Q atoms serve as sites to balance its negative charge through chemical bonding. The high coordination number of rare earth ions precisely matches this unit, allowing one RE cation to bond with one or even multiple [P_2_Q_6_] units. This maximizes the functionality of this basic unit and synergistically enhances the NLO effects in conjunction with [REQ_*n*_] polyhedra. In the following discussion, four systems are introduced: REBS_3_ (RE = La, Ce, Pr, Nd, Sm, and Tb), Ca_2_RE(BS_3_)(SiS_4_) (RE = La, Ce, and Gd), Eu_2_P_2_S_6_, and KREP_2_Se_6_ (RE = Sm, Tb, and Gd).

#### 2.3.1. REBS_3_ (RE = La, Ce, Pr, Nd, Sm, and Tb)

A new class of NLO materials known as thioborates potentially possesses high LIDTs, diverse structures, large *d*_eff_ values, and wide optical transmittance. Among them, REBS_3_ (RE = La, Ce, Pr, Nd, Sm, and Tb) were first to be reported by Hunger *et al.* in 2010, but they required rigorous experimental conditions of high temperature and pressure.^163–166^ Moreover, the samples produced using this method were only polycrystalline powders. Therefore, researchers like Hans-Conrad zur Loye *et al.* explored a new method using the boron–chalcogen mixture approach, with raw materials including RE_2_O_3_/CeO_2_, B, S, and K_2_S/NaI–CsI acting as a flux.^167^ They successfully synthesized REBS_3_ in 2023. In the same year, Mao's group proposed another method to obtain LaBS_3_ by employing La_2_S_3_, B, S, and Li_2_S as a flux and characterized its structure and NLO properties.^168^

**Fig. 16:**
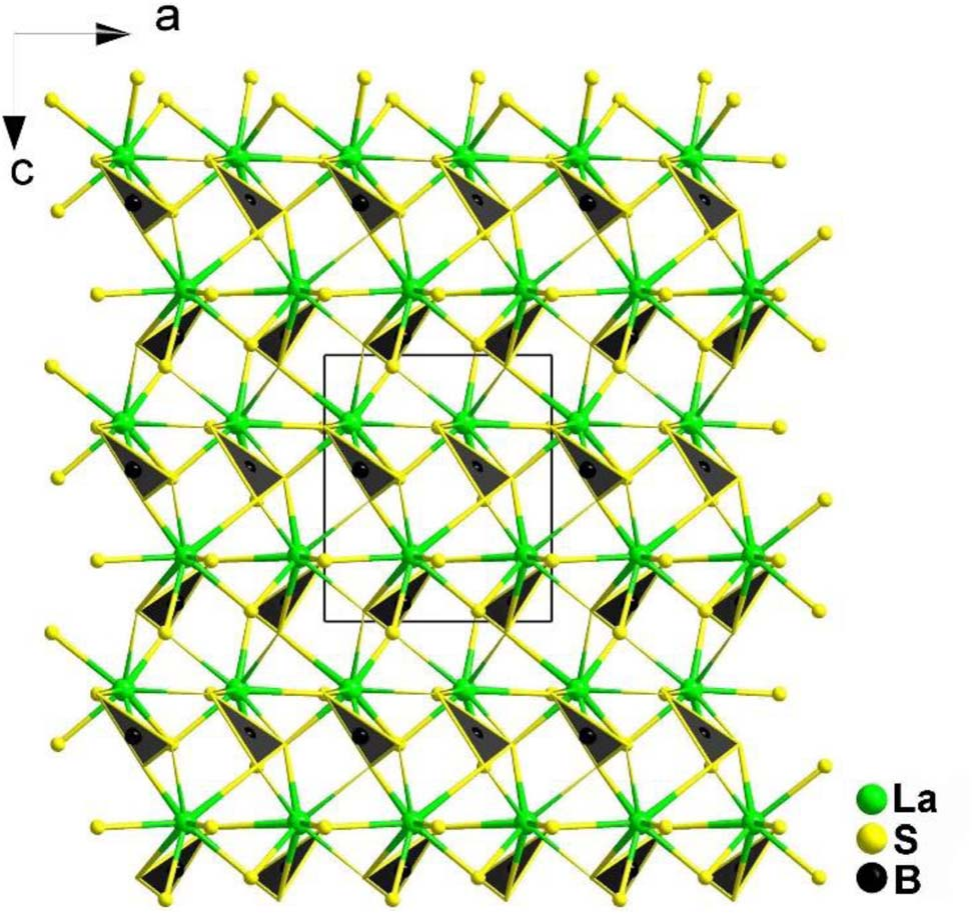
Structure of LaBS_3_ along the *ac* plane with the unit cell outlined.

REBS_3_ (RE = La, Ce, Pr, Nd, Sm, and Tb) are isomorphic and adopt an orthorhombic *Pna*2_1_ space group. LaBS_3_ can serve as an example to discuss the crystal structure; as shown in Fig. 16, its structure comprises triangular [BS_3_]^3−^ units, with each La atom connecting to 9 S atoms to link with the [BS_3_]^3−^ units. Each [BS_3_]^3−^ unit is linked to 6 La^3+^ atoms, ultimately forming the entire 3D network structure.

As expected, LaBS_3_ possesses a large *E*_g_ of 3.18 eV, the highest *d*_eff_ among *ortho*-thioborates (1.2 × AgGaS_2_ at 2050 nm), an ultra-high LIDT of 14 × AgGaS_2_, a wide IR transmission range (0.35–25 μm), and exhibits PM behaviour. The results of theoretical calculations indicate that the [LaS_9_]^15−^ groups and the π-conjugated [BS_3_]^3−^ anions are the main sources of the SHG effect. In general, this research provides us with a new method to synthesize new NLO materials.

#### 2.3.2. Ca_2_RE(BS_3_)(SiS_4_) (RE = La, Ce, and Gd)

Ca_2_RE(BS_3_)(SiS_4_) (RE = La, Ce, and Gd) were synthesized by Mao's group in 2023 using a high-temperature solid-state reaction.^169^ This represents the first case of thioborate–thiosilicates. The researchers employed a mixed flux method, utilizing BaS, CaS, RE_2_S_3_, SiO_2_, B, and S as raw materials.

**Fig. 17:**
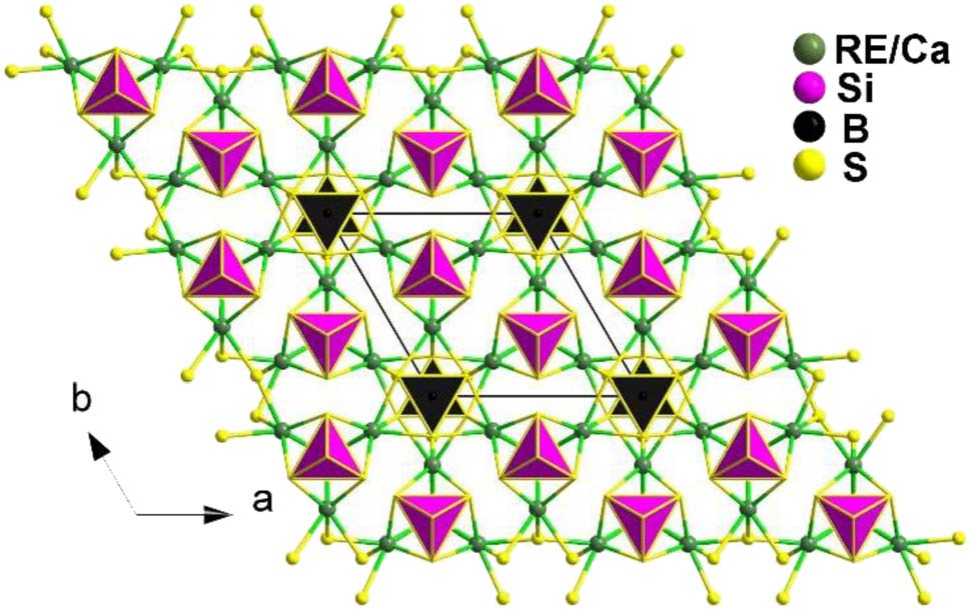
Structure of Ca_2_RE(BS_3_)(SiS_4_) along the *ab* plane with the unit cell outlined.

The compounds Ca_2_RE(BS_3_)(SiS_4_) (RE = La, Ce, and Gd) are isostructural and crystallize in the hexagonal *P*6_3_*mc* space group. Fig. 17 illustrates the structure of Ca_2_RE(BS_3_)(SiS_4_) along the *ab* plane. In this structure, the RE^3+^ and Ca^2+^ ions occupy the same site, referred to as the M site, with a ratio of 1/3 and 2/3, respectively. Each M site coordinates with 6 S atoms to form an [MS_6_] polyhedron, which connects with isolated [BS_3_]^3−^ and [SiS_4_]^4−^ units along the *c* axis, resulting in the overall 3D structure of Ca_2_RE(BS_3_)(SiS_4_).

Remarkably, all of them exhibit comprehensive properties that make them promising candidates for IR-NLO materials. These properties include strong PM *d*_eff_ at 2050 nm (1.1–1.2 × AgGaS_2_), high LIDTs (7–10 × AgGaS_2_), a wide transmission range (0.45–11 μm), high thermal stabilities (>1073 K), and large calculated Δ*n* (0.126–0.149@1064 nm). These properties validate the material design strategy of combining [BS_3_]^3−^ and [SiS_4_]^4−^ NLO-active motifs.

Theoretical calculations indicate that the big *d*_eff_ mainly stem from the synergy between the [RES_6_], [BS_3_], and [SiS_4_] groups. These findings not only expand the scope of research on chalcogenides but also offer a straightforward synthetic method for heteroanionic thioborates.

#### 2.3.3. Eu_2_P_2_S_6_

Compared with common metal chalcogenides, chalcogenophosphates with NCS structures are very few and difficult to synthesize, so they are rarely found as IR-NLO candidate materials. Although the crystal structure of RE-based chalcogenophosphate Eu_2_P_2_S_6_ was reported as early as 1987,^170^ its NLO performance was not reported by the Guo's research group until 2022.^171^

**Fig. 18:**
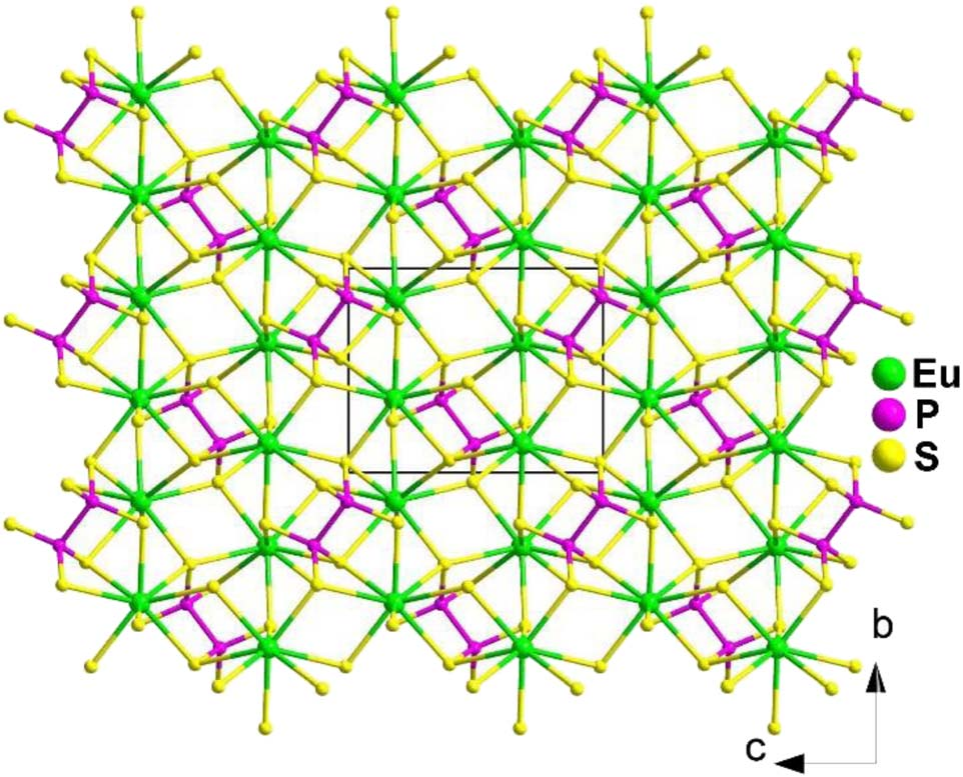
Structure of Eu_2_P_2_S_6_ along the *bc* plane with the unit cell outlined.

Yellow block crystals of Eu_2_P_2_S_6_ were prepared through a high-temperature solid-phase method between stoichiometric Eu, P_2_S_5_, and S at 1233 K. It belongs to the monoclinic *Pn* space group. The structure of Eu_2_P_2_S_6_ can be viewed as comprising bicapped-triangular-prism [EuS_8_] and dimeric [P_2_S_6_] functional primitives built into a 3D framework *via* 8 : 18 : 13 intergrowth (Fig. 18).

This first IR-NLO RE-based chalcogenophosphate, Eu_2_P_2_S_6_, displays wonderful comprehensive optical properties, including a moderate PM *d*_eff_ (0.9 × AgGaS_2_), large LIDT (3.4 × AgGaS_2_), and broad IR transparency region (0.49–15.4 μm). Based on the results of structural analysis and theoretical studies, the overall IR-NLO properties are attributable to the synergetic effect of [EuS_8_] and [P_2_S_6_] functional primitives. This study not only developed the first RE-based chalcogenophosphate as an advanced NLO candidate, but also provided a representative example for the future discovery of high-performance RE-based IR-NLO chalcogenides.

#### 2.3.4. KREP_2_Se_6_ (RE = Sm, Tb, and Gd)

Combining the merits of multiple oxidation states, strong covalent character of the P–Q bonds, and low melting points, Guo's group successfully combined metal chalcophosphates with RE-centered units that exhibit large polarization, resulting in the synthesis of KREP_2_Se_6_ (RE = Sm, Tb, and Gd) in 2022.^172^ The synthesis was carried out using the M_*x*_O_*y*_–B–Q (M = metal; Q = S, Se) solid-state route, which had previously been used to prepare chalcogenides by Huang and Guo *et al.*^108^

**Fig. 19:**
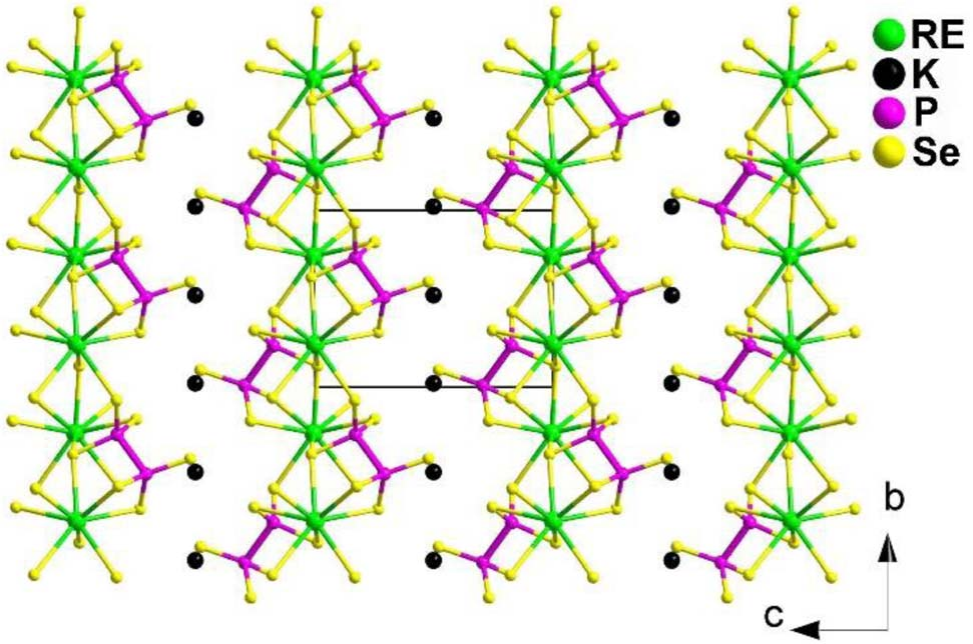
Structure of KREP_2_Se_6_ along the *bc* plane with the unit cell outlined.

The first RE-based selenophosphates KREP_2_Se_6_ (RE = Sm, Tb, and Gd) belong to the monoclinic *P*2_1_ space group. They are composed of a 2D [REP_2_Se_6_]^−^ layer with K^+^ ions filling the spaces in between (Fig. 19). The [REP_2_Se_6_]^−^ layer is built by layers in which each bicapped-trigonal-prism [RESe_8_] unit is linked to four [RESe_8_] units by sharing edges and vertices. These [RESe_8_] units also connect with 4 [P_2_Se_6_]^4−^ units by sharing faces, edges, and corners. The co-vertices are arranged on one side, while the co-edges are arranged on the other.

As a result, KREP_2_Se_6_ (RE = Sm, Tb, and Gd) exhibit a PM *d*_eff_ effect ranging from 0.34 to 1.08 × AgGaS_2_ at 2100 nm and a LIDT ranging from 1.43 to 4.33 × AgGaS_2_. The theoretical calculations suggest that the cooperation between [P_2_Se_6_] and [RESe_8_] plays a predominant role in the *d*_eff_. This research demonstrates that RE-based selenophosphates are a promising type of IR-NLO material. It not only enhances the understanding of crystal chemistry in RE-based chalcophosphates but also expands the range of potential applications for NLO materials.

### 2.4. RE-based chalcohalides and oxychalcogenides

The emergence of heteroanions has enriched traditional functional units, as the differences in electronegativity result in a greater charge distribution shift within the interior of heteroanionic groups compared to conventional functional units.^215–228^ This leads to distortions and the display of significant static dipole moments and anisotropy.^229–233^ Unlike traditional chalcogenides, the presence of heteroanionic groups combines the high polarizability of chalcogenides with the advantages of oxides and halides that have wide *E*_g_. RE-based chalcohalides and oxychalcogenides not only incorporate disparate anions but also feature the presence of salt-inclusion compounds. Below, eleven RE-based chalcohalides and oxychalcogenides are introduced.

#### 2.4.1. La_6_Cd_0.75_Ga_2_Q_11.5_Cl_2.5_ (Q = S, Se)

**Fig. 20:**
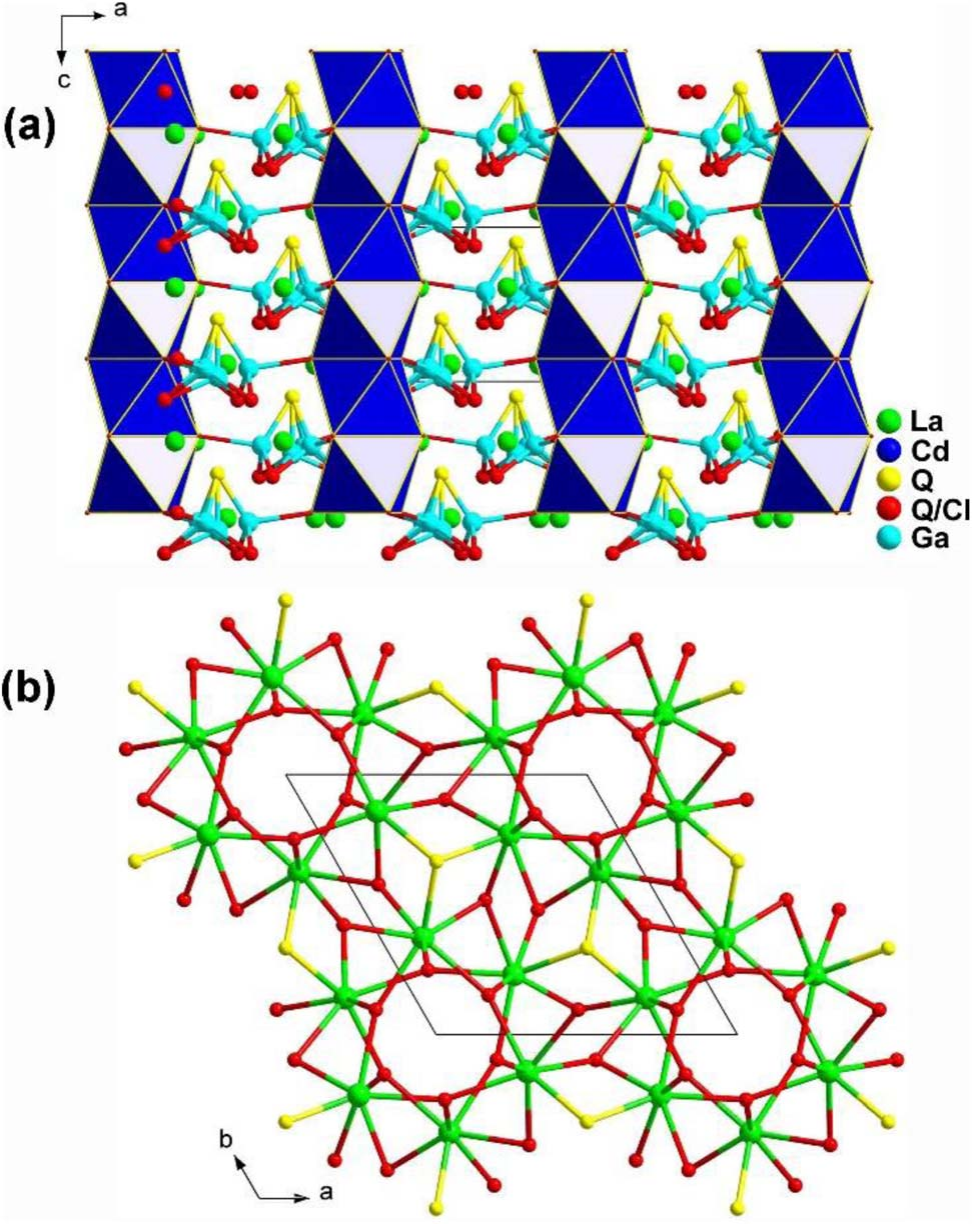
Structure of (a) La_6_Cd_0.75_Ga_2_Q_11.5_Cl_2.5_ along the *ac* plane and (b) 3D La/Q/(Q/Cl) network along the *ab* plane with the unit cell outlined.

Two novel quinary chalcohalides, La_6_Cd_0.75_Ga_2_Q_11.5_Cl_2.5_ (Q = S, Se), with heteroanionic functional motifs were discovered in 2021 by Huang's group using LaCl_3_ as a reaction-flux.^173^ Both compounds belong to the NCS space group *P*6_3_. Fig. 20a displays a schematic diagram of La_6_Cd_0.75_Ga_2_Q_11.5_Cl_2.5_ seen along the *ac* plane. In the structure, plane-linked 1D [Cd(Q/Cl)_6_] chains are packed along the 6_3_ axes, and the tetrahedral [GaQ_4_] units are separated along the direction of the 3-fold rotation axes. Furthermore, the [La(Q/Cl)_6_Q] polyhedra form a 3D network structure in the *ab* plane through vertex- and edge-sharing as shown in Fig. 20b with the unit cell outlined.

Moreover, the introduction of various heteroanionic functional motifs with varying sizes and electronegativity increases the distortion of structural groups while keeping the structural symmetry. This enhances the polarity of the NLO-active motifs, resulting in a greater *d*_eff_. As expected, La_6_Cd_0.75_Ga_2_S_11.5_Cl_2.5_ exhibits a large LIDT of 18.6 × AgGaS_2_ @1064 nm and a strong *d*_eff_ of 0.8 × AgGaS_2_ @43–75 μm under 2050 nm laser irradiation.

#### 2.4.2. RE_3_AsS_5_X_2_ (RE = La, Pr; X = Cl, Br)

The NCS chalcohalide La_3_AsS_5_Br_2_ with the monoclinic space group *Cc* (no. 9),^174^ which is isostructural to the previously reported Pr_3_AsS_5_Cl_2_,^175^ was successfully prepared using a salt flux growth method in 2023 by Wang's group.

**Fig. 21:**
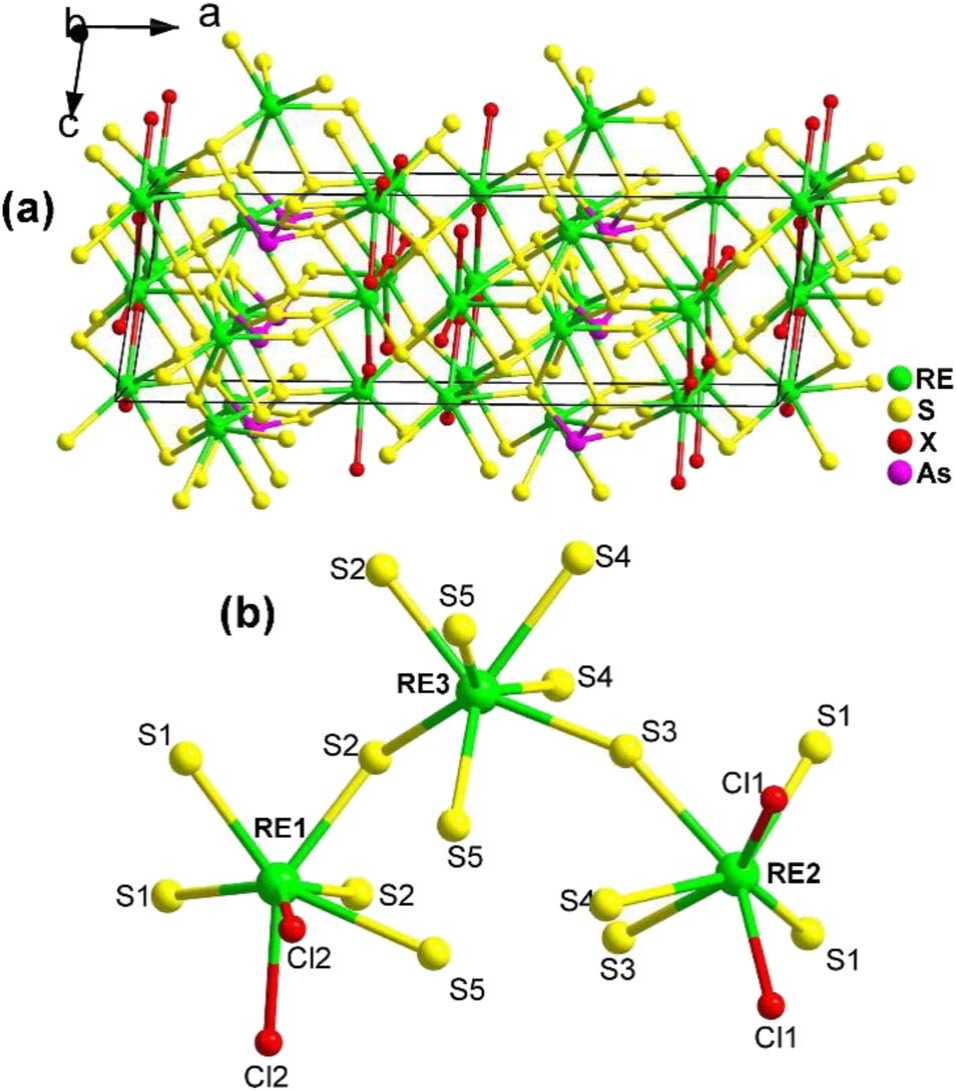
Structure of (a) RE_3_AsS_5_X_2_ along the *ac* plane with the unit cell outlined and (b) coordination environment of the crystallographically independent RE atoms.

The 3D network of RE_3_AsS_5_X_2_ (RE = La, Pr; X = Cl, Br) is composed of [RE1S_5_X_3_] bicapped trigonal prisms, [RE2S_5_X_3_] bicapped trigonal prisms, [RE3S_7_] capped trigonal prisms, and SACLP [AsS_3_] trigonal pyramids, which are interconnected with each other (Fig. 21a). The coordination environment of the crystallographically independent RE atoms in the structure of RE_3_AsS_5_X_2_ is provided in Fig. 21b. The electron localization function results confirmed that the arrangement of the [AsS_3_] groups contributes to the NCS nature of RE_3_AsS_5_X_2_.

The moderate PM *d*_eff_ (0.23 × AgGaS_2_ @150–210 μm), easy growth of millimeter-sized crystals, excellent ambient stability, and wide *E*_g_ (*ca.* 2.9 eV) of La_3_AsS_5_Br_2_ suggest its potential applicability as an IR-NLO candidate.

#### 2.4.3. Eu_4.5_(B_5_O_9_)_2_SI

The first sulfide borate to possess NLO activity, Eu_4.5_(B_5_O_9_)_2_SI, contains I^−^, S^2−^, and borate anions simultaneously. It was synthesized by Guo's group in 2019 as a derivative of Eu_2_B_5_O_9_S using KI as a flux in a solid-state reaction.^176^ Both Eu_4.5_(B_5_O_9_)_2_SI and Eu_2_B_5_O_9_S crystallize in the NCS orthorhombic *Pnn*2 space group, but Eu_2_B_5_O_9_S does not exhibit apparent SHG. Here, we discuss the structure of Eu_4.5_(B_5_O_9_)_2_SI in detail.

**Fig. 22:**
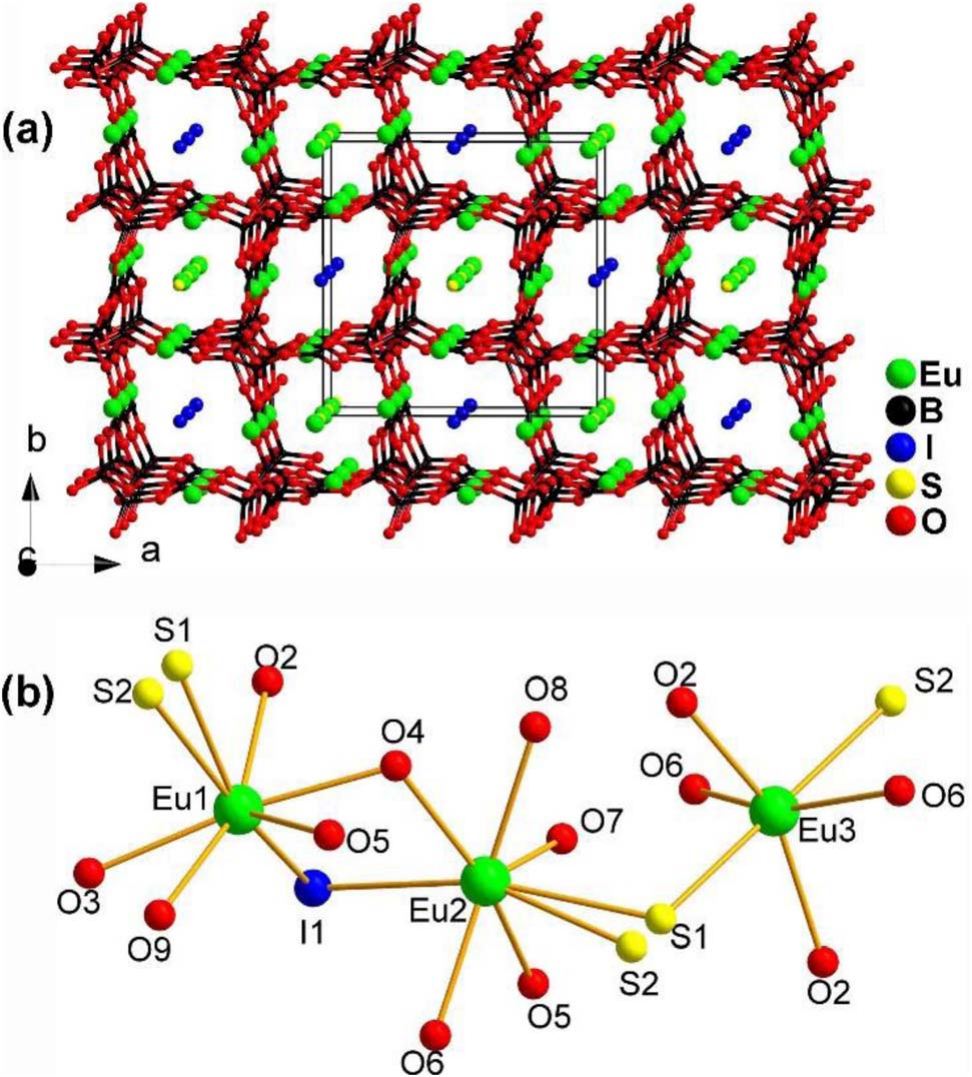
Structure of (a) Eu_4.5_(B_5_O_9_)_2_SI along the *ab* plane with the unit cell outlined and (b) coordination environment of the crystallographically independent Eu atoms.

The structure of Eu_4.5_(B_5_O_9_)_2_SI along the *ab* plane is displayed in Fig. 22a. It consists of a 3D polyanionic network {(B_5_O_9_)^3−^}_∞_, which is constructed by linking 3 [BO_4_] tetrahedra with 2 [BO_3_] planar triangles through shared O atoms. Along the *c* axis, there is a tubular accumulation [Eu_2_(B_5_O_9_)]_∞_ framework built by Eu_1_ and Eu_2_ located in the cavities of the 3D network. The cavities of the 3D network are also occupied by I^−^ ions. Fig. 22b shows the coordination environment of the crystallographically independent Eu atoms, which have three different coordination modes: [Eu_1_O_5_S_2_I], [Eu_2_O_5_S_2_I], and [Eu_3_O_4_S_2_].

The combination of Eu^2+^, S^2−^, I^−^, and borates allows for the realization of a large *d*_eff_ and a high LIDT. Eu_4.5_(B_5_O_9_)_2_SI has an *E*_g_ of 1.99 eV, a moderate *d*_eff_ of 0.5 × AgGaS_2_, a high LIDT of 15 × AgGaS_2_, and PM behaviour. These results indicate that the Eu–S and B–O motifs play an important role in the *d*_eff_ and offer an example of the practicality of this strategy.

#### 2.4.4. (K_3_I)[REB_12_(GaS_4_)_3_] (RE = Sm, Gd)

**Fig. 23:**
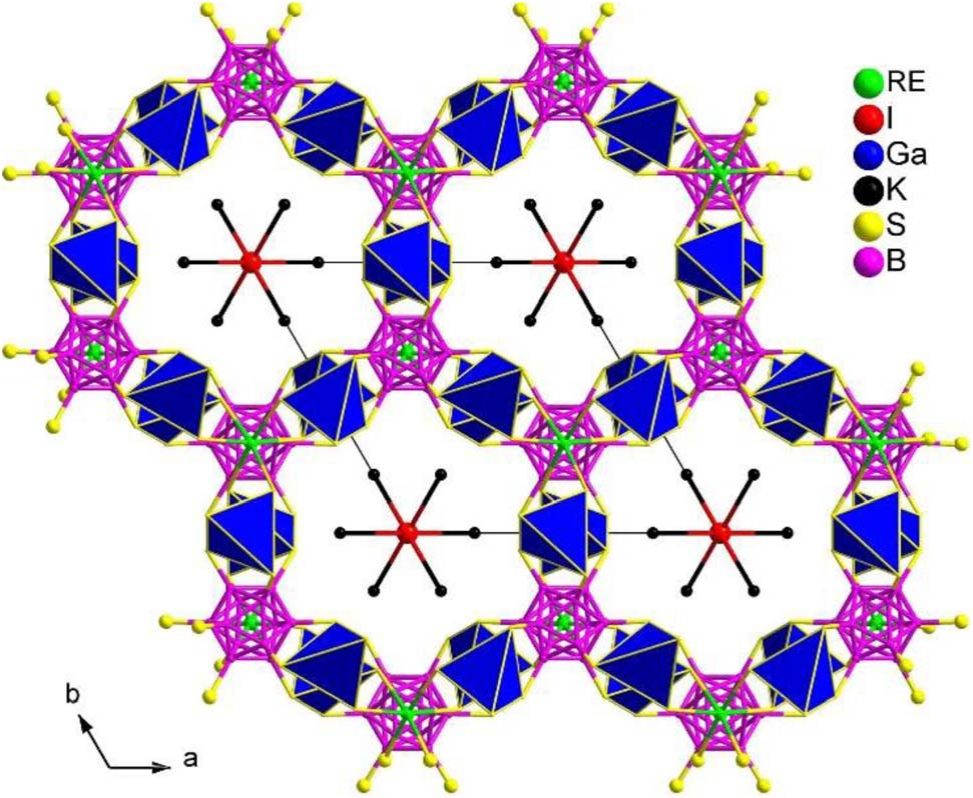
Structure of (K_3_I)[REB_12_(GaS_4_)_3_] with the unit cell outlined along the *ab* plane.

The crystal structure of (K_3_I)[SmB_12_(GaS_4_)_3_], which was reported by Guo's group in 2009, represents the first salt-inclusion chalcogenide with NLO activity.^177^ Throughout the next decade, multiple isomorphic compounds were synthesized using high-temperature solid-phase methods and various alkali-metal halide fluxes, with B being used in its original form.^234–236^

(K_3_I)[REB_12_(GaS_4_)_3_] (RE = Sm, Gd) share the same structure and belong to the hexagonal chiral space group *P*6_3_22. In the asymmetric unit, there are 1 A, 1 X, 1 RE, 1 Ga, 2 B, and 2 Q positions. The crystal structure consists of two parts: the polycationic [K_6_I]^5+^ and the 3D open [REB_12_(GaS_4_)_3_]^5−^ anionic framework. The 3D framework comprises icosahedral [B_12_], tetrahedral [GaS_4_], and octahedral [RES_6_] functional primitives. Within the structure, the [B_12_] icosahedra are surrounded by 6 [GaS_4_] tetrahedra and consolidated by 2 [RES_6_] octahedra, resulting in a 3D honeycomb-like open framework. This framework forms channels occupied by face-sharing [IK_6_] octahedral chains along the *c* direction (see Fig. 23).

Due to the significantly low yield for most compounds, the majority of samples in this system were unable to undergo size-dependent SHG measurements. For instance, the *d*_eff_ of (K_3_I)[SmB_12_(GaS_4_)_3_] is approximately 0.3 times that of KDP when subjected to a 1940 nm laser. The small *d*_eff_ can be attributed to the arrangement of the SHG-active groups in their structures, which hinders the improvement of macroscopic polarizabilities.

#### 2.4.5. [RE_3_S_2_Cl_2_][SbS_3_] (RE = La, Ce) and [La_3_OSCl_2_][SbS_3_]

RE-based chalcohalide [RE_3_S_2_Cl_2_][SbS_3_] (RE = La, Ce) and oxychalcogenide [La_3_OSCl_2_][SbS_3_] were reported by Zhao's research group in 2021^178^ and 2022,^179^ respectively. Although they show identical stoichiometry, they belong to different crystal systems and exhibit different structural symmetries.

**Fig. 24:**
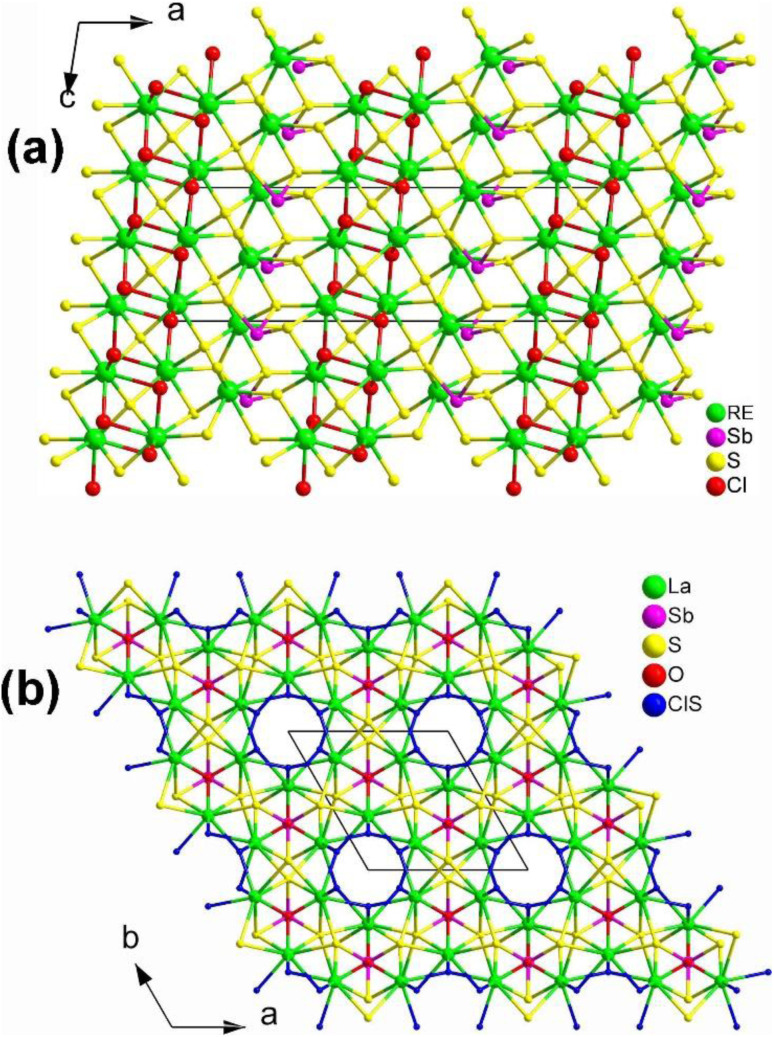
Structures of (a) [RE_3_S_2_Cl_2_][SbS_3_] along the *ac* plane and (b) [La_3_OSCl_2_][SbS_3_] along the *ab* plane with unit cell outlined.

[RE_3_S_2_Cl_2_][SbS_3_] (RE = La, Ce) were obtained from a mixture of CsCl, RE, GaCl_3_, SbCl_3_, and S in a ratio of 1 : 4 : 1 : 1 : 5 using a high-temperature solid-state method at 1073 K. As shown in Fig. 24a, [RE_3_S_2_Cl_2_][SbS_3_] belongs to the NCS *Cc* space group, where the 2D layers of [RE_3_S_2_Cl_2_]^12+^ arranged in the *bc* plane and the trigonal-pyramid [SbS_3_] motifs are stacked in an alternating pattern. However, the stacking of [SbS_3_] motifs in the crystal cell is in-phase and they are correlated with each other through the *n* sliding planes at (*x*, 1/4, *z*). Unfortunately, no NLO performance has been reported for these two compounds.

Yellow crystals of [La_3_OSCl_2_][SbS_3_] were grown from a mixture of La, LaCl_3_, Sb, Sb_2_O_3_, and S with a ratio of 7 : 2 : 1 : 1 : 12 at 1223 K. It can be seen as being obtained through partial isovalent anion substitution of the parent structure [La_3_S_2_Cl_2_][SbS_3_]. Interestingly, [La_3_OSCl_2_][SbS_3_] adopts the hexagonal space group *P*6_3_*mc*, which is not isostructural with [La_3_S_2_Cl_2_][SbS_3_] (monoclinic space group *Cc*), despite their having similar functional primitives. [La_3_OSCl_2_][SbS_3_] is made of 3D cationic [La_3_OSCl_2_]^3+^ frameworks hosting covalent [SbS_3_]^3−^ motifs. The 3D cationic [La_3_OSCl_2_]^3+^ are built *via* the 1D hexagonal columns of corner-sharing [(Cl/S)La_3_] polyhedra propagating along the 6_3_ axis situated in (0, 0, *z*) and [OLa_3_] trigonal pyramids through vertex-sharing. It is worth mentioning that experimental results indicate a moderate *d*_eff_ (*ca.* 0.7 × AgGaS_2_@50–100 μm) under a 2050 nm laser, and the calculated Δ*n* value is about 0.27 at 2050 nm, respectively.

#### 2.4.6. YSeBO_2_

Due to the difficulty of their synthesis, chalcogenide borates have been rarely studied as IR-NLO candidate materials in the past. The second selenide borate, YSeBO_2_, was successfully discovered by Guo's group in 2020.^180^ Transparent rodlike crystals of YSeBO_2_ with a large *E*_g_ of 3.45 eV were prepared through a traditional solid-phase reaction between Y_2_O_3_, B_2_O_3_, B, and Se and additional KI used as the flux.

**Fig. 25:**
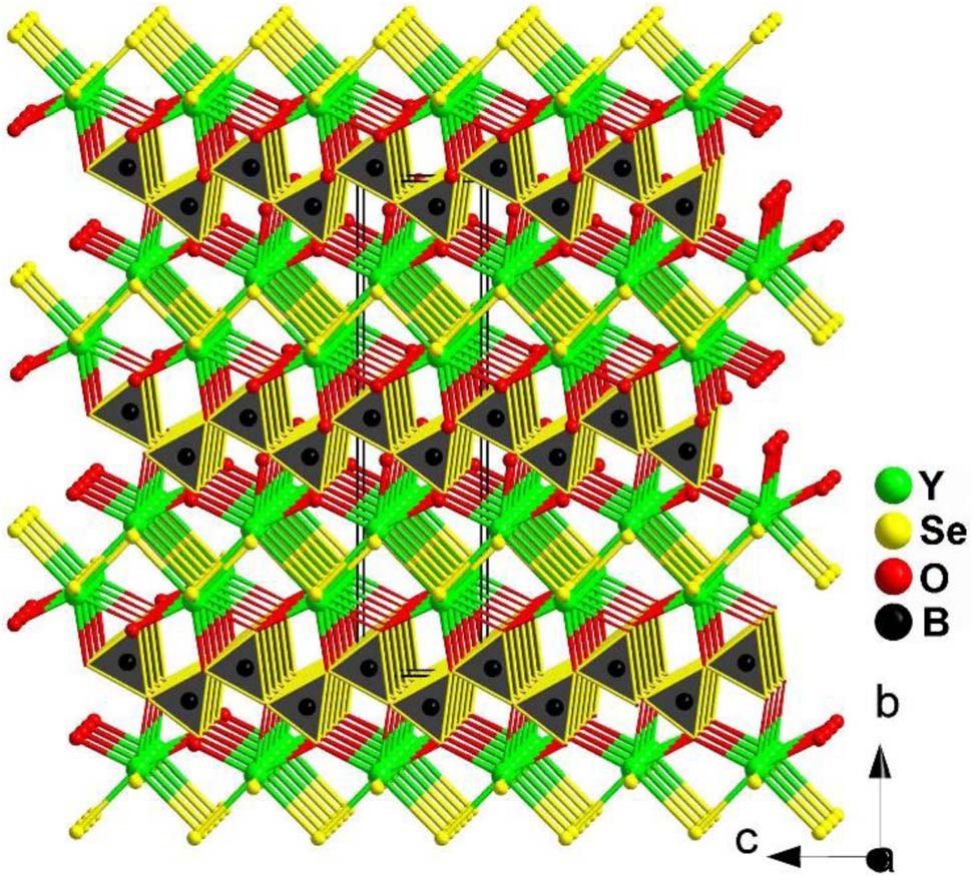
Structure of YSeBO_2_ along the *bc* plane with the unit cell outlined.

YSeBO_2_ adopts the orthorhombic polar space group *Cmc*2_1_, and the 3D structure is formed by the connection between [YO_3_Se_4_]^11−^ pentagonal bipyramids and [BO_3_]^3−^ planar triangles by sharing O edges (see Fig. 25). It is worth mentioning that the heteroanionic [YO_3_Se_4_]^11−^ functional motif was first discovered in the chalcogenide system. In addition, YSeBO_2_ displays a weak *d*_eff_ of about 0.2 × KDP (@150–210 μm) with PM features. Theoretical analysis shows that the Se-4p and Y-4d states play a major role in the origin of SHG. The contribution of the [BO_3_]^3−^ planar triangles did not show any impact due to its unfavorable arrangement.

#### 2.4.7. Eu_3_GeOS_4_

Using a mixed-anion strategy, Guo's group successfully obtained the first quaternary RE-based oxythiogermanate Eu_3_GeOS_4_ by introducing Eu and [GeOS_3_] in 2023.^181^ The millimeter-size crystals of Eu_3_GeOS_4_ were prepared by using a traditional solid-state method at 1148 K.

**Fig. 26:**
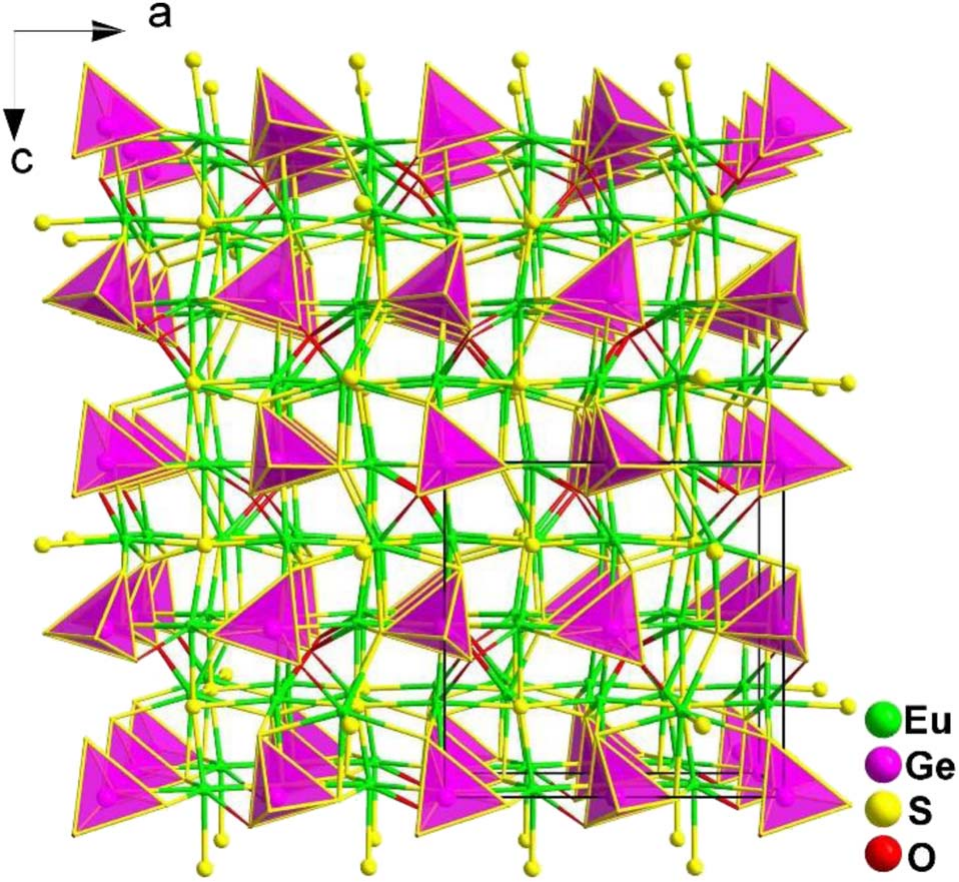
Central projection of Eu_3_GeOS_4_ along the *ac* plane with the unit cell outlined.

Eu_3_GeOS_4_ belongs to the orthorhombic *Pca*2_1_ space group. As displayed in Fig. 26, neighbouring [EuOS_6_] mono-capped trigonal prisms are interlinked through sharing faces to form a 3D framework structure, with isolated [GeOS_3_] tetrahedra occupying the cavities. In other words, without considering the Eu–O/S bonds, the crystal structure of Eu_3_GeOS_4_ can also be considered a pseudo-0D structure. It is interesting to note that the coordination mode of the heteroanionic [EuOS_6_] functional motif was discovered for the first time in oxychalcogenides.

Eu_3_GeOS_4_ not only shows an obvious *d*_eff_ (0.24 × AgGaS_2_@2050 nm), but also a large LIDT (8.86 × AgGaS_2_@1064 nm). Theoretical calculations indicate that the *d*_eff_ is attributed to the synergistic effect of the heteroanionic [EuOS_6_] and [GeOS_3_] functional motifs.

#### 2.4.8. RE_3_NbS_3_O_4_ (RE = Ce, Sm, Gd, Dy)

A series of RE-based oxysulfides RE_3_NbS_3_O_4_ (RE = Ce, Sm, Gd, Dy) were systemically investigated. Among them, the Sm and Gd compounds display NCS structures (space group: *Pna*2_1_), while the Ce and Dy compounds exhibit CS structures (space group: *Pnma*), which is mainly caused by the various coordination types and stacking direction of Nb atoms as well as the different ionic radii of RE^3+^ cations.^182–185^

**Fig. 27:**
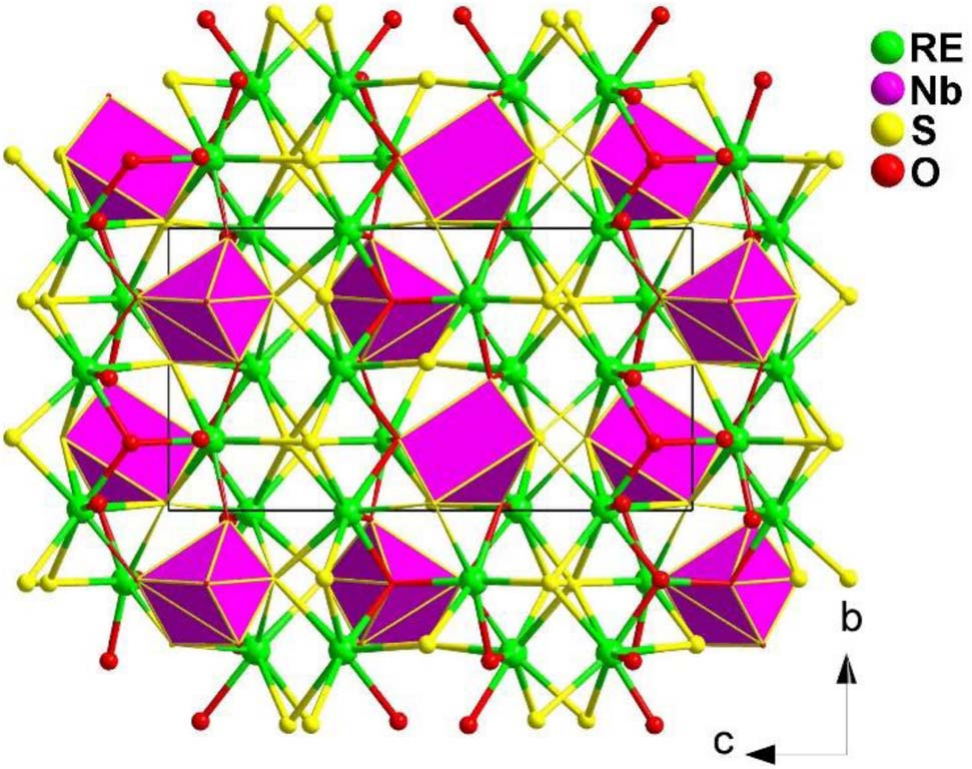
Structure of RE_3_NbS_3_O_4_ along the *bc* plane with the unit cell outlined.

The crystals of RE_3_NbS_3_O_4_ were prepared through solid-phase reactions at 1273 K using RE_2_O_3_, Nb_2_O_5_, B, and S as the raw materials and excess KI as the flux. When charge-balanced RE^3+^ cations are omitted, the NCS structure of RE_3_NbS_3_O_4_ (RE = Sm, Gd) can be seen as a hexagonal cage formed by 6 Nb atoms encasing 6 RE atoms when viewed along the *bc* plane (see Fig. 27). Each RE forms two different coordination forms: a [RES_4_O_4_] double-capped triangular prism and a [RES_5_O_3_] dodecahedron.

Sm_3_NbS_3_O_4_ and Gd_3_NbS_3_O_4_ exhibit PM *d*_eff_ of about 0.3 and 0.4 × AgGaS_2_ under a 2100 nm laser and large LIDTs of 12.5 and 4.5 × AgGaS_2_ at 1064 nm. The heteroanionic [NbS_2_O_4_] unit was first found as a SHG-active motif, which offers a new route for discovering novel IR-NLO oxychalcogenides.

#### 2.4.9. Nd_3_[Ga_3_O_3_S_3_][Ge_2_O_7_]

Previous studies have shown that it is challenging to achieve chalcogenides based on rare earth (RE) materials that have both a large band gap (*E*_g_ > 3.5 eV) and a high effective nonlinear optical coefficient (*d*_eff_ > 0.5 × AgGaS_2_). However, in 2023, the Zhu research group successfully designed and synthesized a new RE-based oxychalcogenide, Nd_3_[Ga_3_O_3_][Ge_2_O_7_], for the first time.^186^ This was achieved through a module substitution strategy using the parent structure Cs_3_[Sb_3_O_6_][Ge_2_O_7_].

**Fig. 28:**
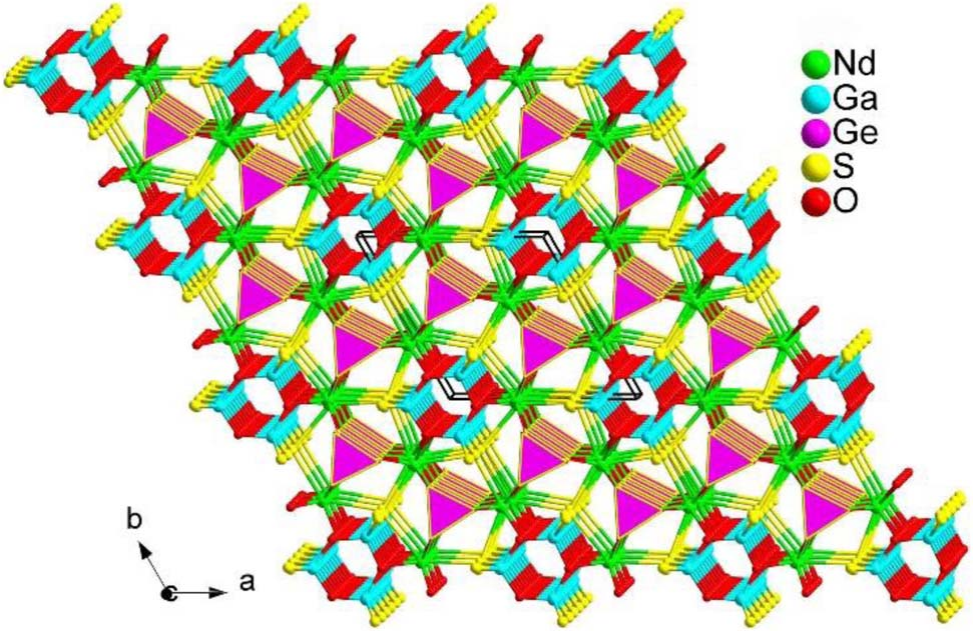
Structure of Nd_3_[Ga_3_O_3_S_3_][Ge_2_O_7_] along the *ab* plane with unit the cell outlined.

Nd_3_[Ga_3_O_3_][Ge_2_O_7_] was discovered using the solid-phase method at 1273 K and adopts the hexagonal space group *P*6̄2*c* (no. 190). Its unique structure consists of three different modules: charge-balanced Nd^3+^ cations, 0D [Ge_2_O_7_]^6−^ dimers, and 1D [Ga_3_O_3_S_3_]^3−^ tubular chains (Fig. 28). The Nd^3+^ cation is coordinated with O/S atoms to form a heteroligand [NdO_6_S_2_] polyhedron. Experimental investigations of Nd_3_[Ga_3_O_3_][Ge_2_O_7_] demonstrate that it is the first RE-based IR-NLO oxychalcogenide, exhibiting excellent optical performance. This includes a strong *d*_eff_ (*ca.* 0.8 × AgGaS_2_@2050 nm), a large *E*_g_ (*ca.* 4.35 eV) corresponding to an ultrahigh LIDT of 23 × AgGaS_2_@1064 nm, a wide transparent window (0.25–13.7 μm), and good thermal stability (<1200 K).

Detailed theoretical results indicate that the remarkable *d*_eff_ in Nd_3_[Ga_3_O_3_][Ge_2_O_7_] is mainly attributed to the cooperation of heteroanionic [GaO_2_S_2_] and [NdO_2_S_6_] functional motifs. This research not only expands the possibilities of IR-NLO families with heteroanionic motifs, but also offers a feasible strategy for the development of other high-performance IR-NLO systems.

#### 2.4.10. Eu_2_M^II^Ge_2_OS_6_ (M^II^ = Mn, Fe, and Co)

By coupling RE elements with localized f-electrons, d-block transition metals with delocalized d-electrons, and the mixed anion group [GeOS_3_] with high polarizability, Guo's group successfully obtained the first melilite-type RE-based oxythiogermanates, Eu_2_M^II^Ge_2_OS_6_, where M^II^ represents Mn, Fe, and Co, in 2023. These compounds were prepared *via* high-temperature solid-phase synthesis using KI as a flux.^187^

**Fig. 29:**
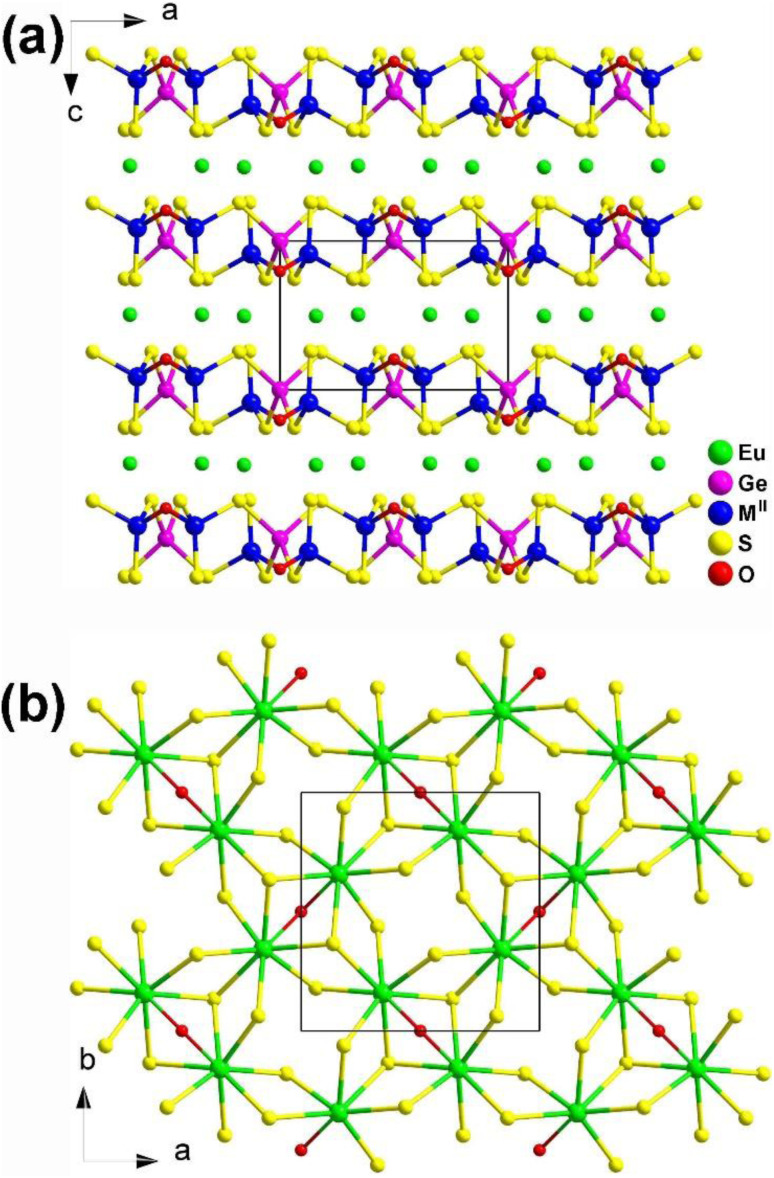
Structure of (a) Eu_2_M^II^Ge_2_OS_6_ along the *ac* plane and (b) the 2D Eu/O/S network along the *ab* plane with the unit cell outlined.

Eu_2_M^II^Ge_2_OS_6_ (M^II^ = Mn, Fe, and Co) are isomorphic and grow in a tetragonal NCS *P*4̄2_1_*m* space group. As shown in Fig. 29a, two neighboring [GeOS_3_] tetrahedra build a dimer, [Ge_2_OS_6_], by sharing an O atom. Each [Ge_2_OS_6_] dimer then interlinks with 4 [M^II^S_4_] tetrahedra by sharing S corners, constructing corrugated 2D [M^II^Ge_2_OS_6_]^4−^ layers along the *ab* plane. The [EuOS_7_] bicapped trigonal prisms are located within these 2D interlayers and serve to bridge adjacent [EuOS_7_] together, forming the 3D network (Fig. 29b).

As a result, Eu_2_M^II^Ge_2_OS_6_ (M^II^ = Mn, Fe, and Co) display excellent IR-NLO performance with an *E*_g_ of 2.11–2.40 eV, a PM *d*_eff_ of 0.3–0.5 × AgGaS_2_ at 200–250 μm, and a large LIDT of 2.8–8.3 × AgGaS_2_. They proposed that these compounds have the potential to replace M^II^ using Cu or other d-block metals and other substitutions within the same family.

#### 2.4.11. REAEGa_3_S_6_O (RE = La, Pr, and Nd; AE = Sr, Ca)

Recent studies have mainly focused on the individual performances of oxysulfides, but there has been little investigation into the comparisons in the specific properties and changes in a series of analogues including oxides, sulfides, and oxysulfides. In order to address this gap, a series of REAEGa_3_S_6_O (RE = La, Pr, and Nd; AE = Sr, Ca) compounds were synthesized by Wu and co-works in 2022, along with their corresponding LaAEGa_3_O_7_ oxides and LaAEGa_3_S_7_ sulfides, which served as reference materials.^128^ The aim was to systematically study the trends in the key properties from oxides to sulfides to oxysulfides.

**Fig. 30:**
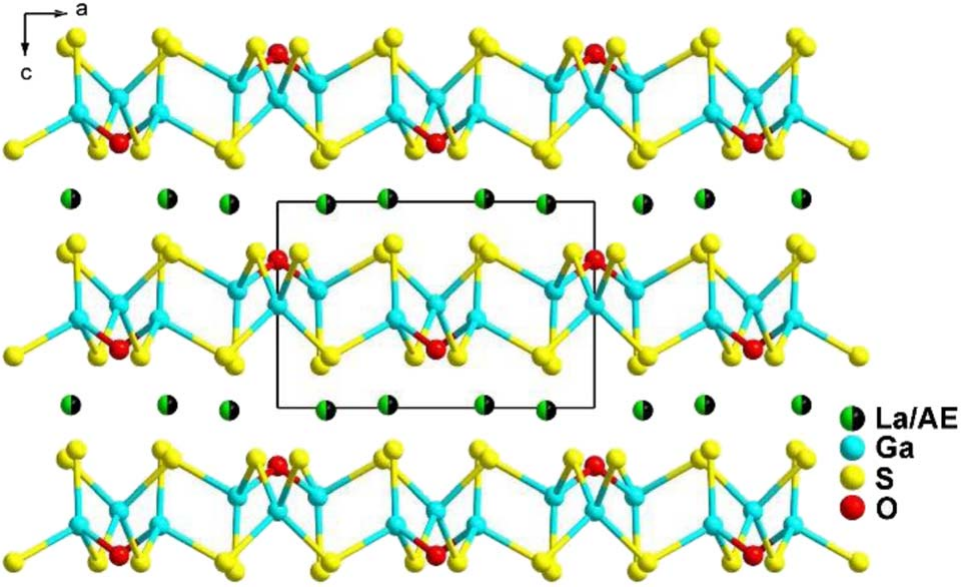
Structure of LaAEGa_3_S_6_O along the *ac* plane with the unit cell outlined.

All of the REAEGa_3_S_6_O oxysulfides belong to the *P*4̄2_1_*m* space group of the tetragonal system, and they are isostructural with LaAEGa_3_O_7_ and LaAEGa_3_S_7_. Fig. 30 presents the unique structure of these compounds, which can be described as consisting of two functional modules: charge-balanced [RE/AE]^5+^ cations and 2D [Ga_3_S_6_O]^5−^ Cairo pentagonal layers. The charge-balanced module is composed of [(RE/AE)S_7_O] groups that are connected through edge- and face-sharing interactions, forming a 2D [(RE/AE)S_7_O]_*n*_ layer. On the other hand, the 2D layers can be seen as a combination of many 5-membered rings, with two [GaS_4_] and three [GaS_3_O] units surrounding each ring.

In particular, REAEGa_3_S_6_O fulfills the property balance requirements (*e.g.*, wide *E*_g_: 3.21–3.27 eV and PM *d*_eff_: 0.9–1.0 × AgGaS_2_). This compound shows promise as a potential IR-NLO crystal, as it combines the advantages of LaAEGa_3_O_7_ and LaAEGa_3_S_7_ through heteroanion-oriented performance engineering. The structure–activity relationships reveal that the heteroanionic [(RE/AE)S_7_O] and [GaS_3_O] functional motifs are particularly promising for NLO activity, as they exhibit large cooperation in the origin of SHG. These findings not only provide a clear understanding of the variation in properties from oxide to sulfide to oxysulfide, but also highlight the feasibility of using heteroanion-oriented design to develop novel NLO candidates with well-balanced performances.

## 3. Conclusions and perspectives

**Scheme 1:**
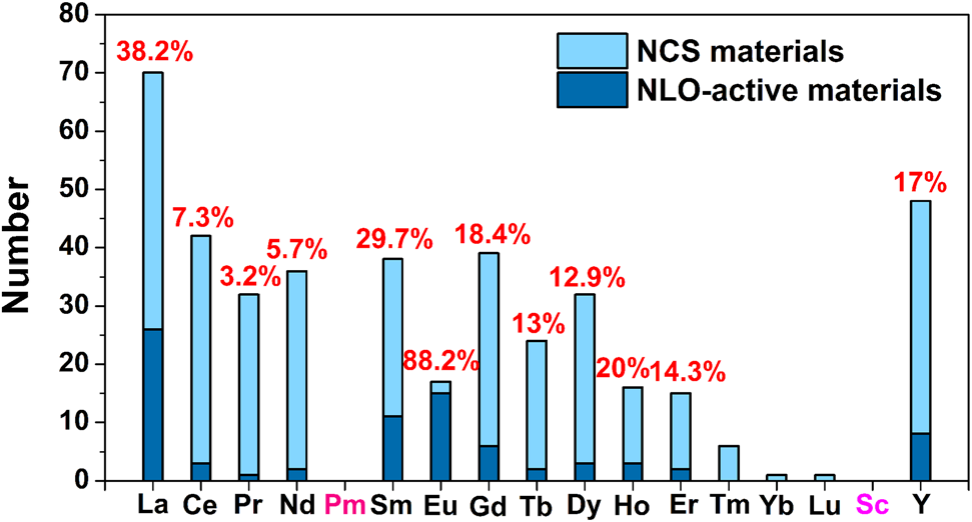
Distribution and proportion of NLO activity in different RE-based NCS materials.

**Scheme 2:**
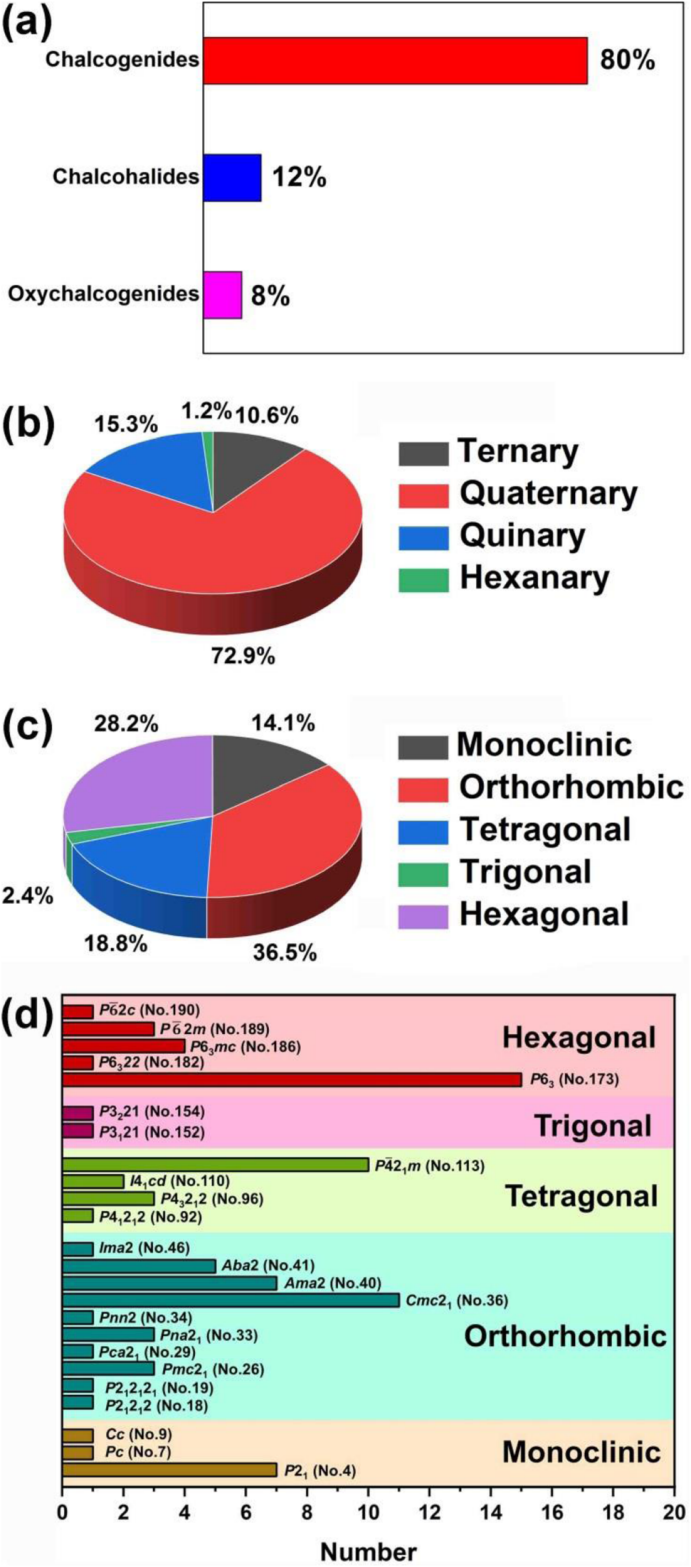
Distributions of RE-based IR-NLO materials based on (a and b) chemical composition, (c) crystal system, and (d) the space groups in five crystal systems.

**Scheme 3:**
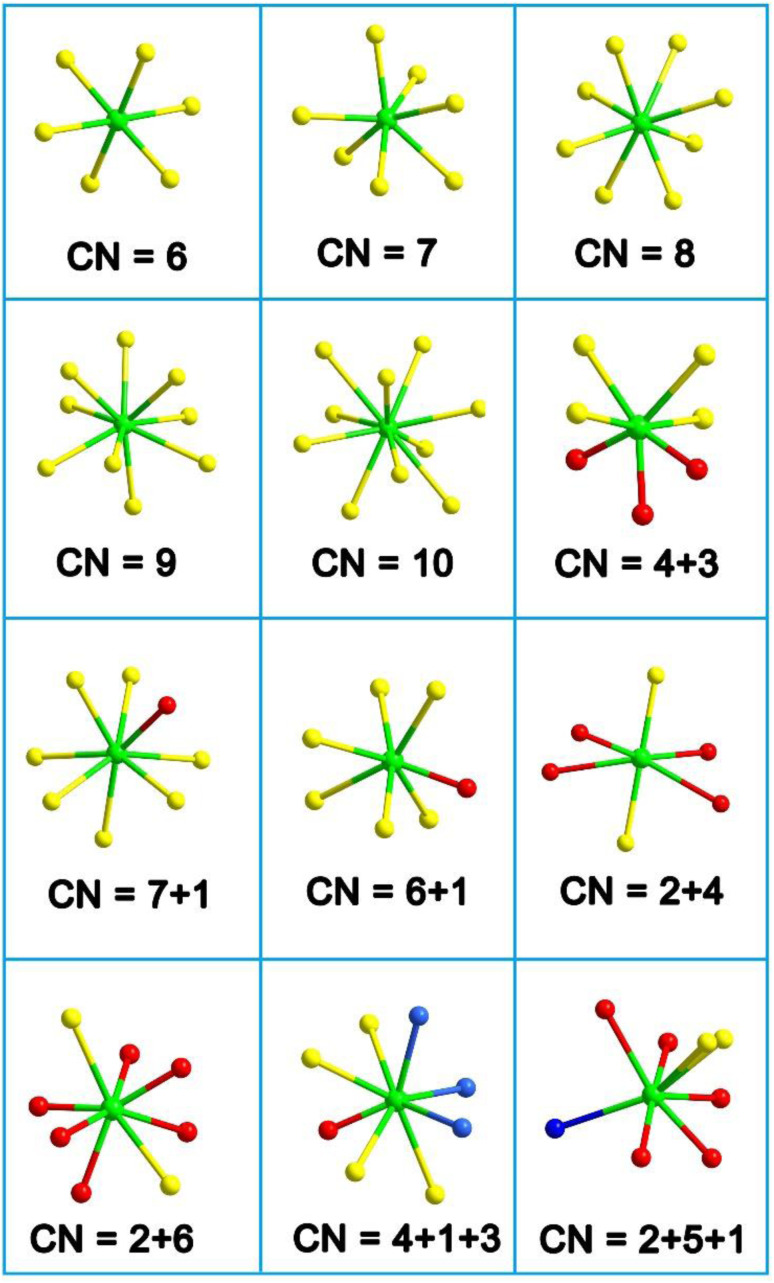
Coordination modes of RE-based asymmetric building motifs (CN = coordination number). Green atom: RE; yellow atom: chalcogen (Q); red atom: O; blue atom: halogen (X).

**Scheme 4:**
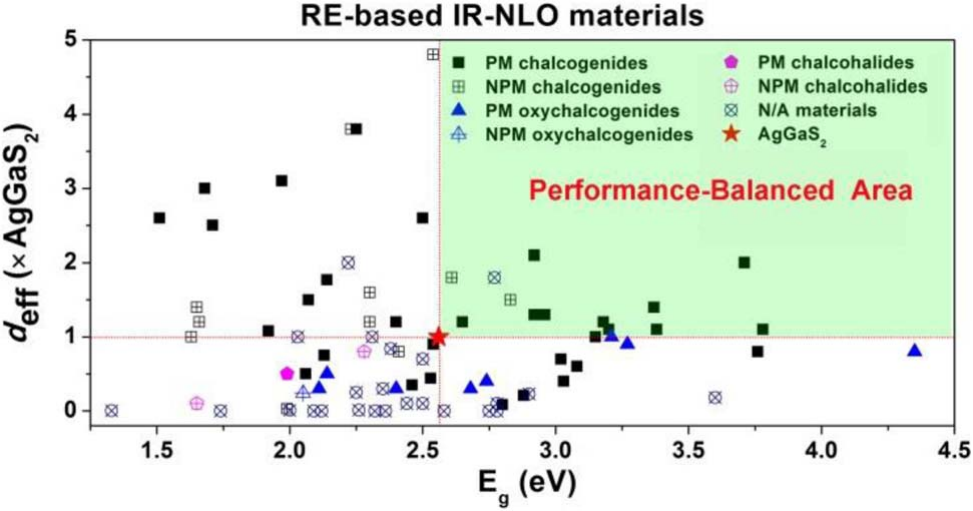
Comparison of the *d*_eff_ (×AgGaS_2_) and *E*_g_ (eV) for selected RE-based materials with IR-NLO properties (the green shaded region represents the performance-balanced area, wherein *d*_eff_ > 1.0 AgGaS_2_ and *E*_g_ > 2.56 eV).

RE-based chalcogenides, with their unique electronic configurations, have been extensively discussed in the field of IR-NLO for several decades, and there has been growing interest in recent years. According to incomplete statistics, there are over 400 compounds with non-centrosymmetric structures among RE-based chalcogenides. However, to date, there has not been a comprehensive review that systematically summarized these compounds. Therefore, this paper provided a summary analysis of their synthesis methods, structures, optical properties, and structure–property relationships. The results of the statistical analysis of these compounds are summarized in Tables 1–4 and Schemes 1–4. Additionally, some key findings have been uncovered:

**Table 1:** Summary of reported RE-based chalcogenides containing tetrahedral motifs

Compound	[REQ_*n*_] polyhedra	Space group	*E*_g_ a (eV)	*d*_eff_ b (×AgGaS_2_)	LIDT^b^ (×AgGaS_2_)	NPM/PM^c^	Δ*n*^d^	Maximum temperature^f^	Ref.
Dy_3_GaS_6_	[DyS_7_]	*Cmc*2_1_ (no. 36)	2.80	0.084	14	PM	0.068	1223 K	69
Y_3_GaS_6_	[YS_7_]	*Cmc*2_1_ (no. 36)	2.88	0.21	18	PM	0.069	1223 K	69
Ho_3_GaS_6_	[HoS_7_]	*Cmc*2_1_ (no. 36)	3.03	0.4	N/A^e^	PM	0.041	1223 K	70
Er_3_GaS_6_	[ErS_7_]	*Cmc*2_1_ (no. 36)	3.08	0.6	N/A	PM	0.034	1223 K	70
Eu_8_Sn_4_Se_20_	[EuSe_9_]	*P*2_1_2_1_2 (no. 18)	1.33	1 × α-SiO_2_@150–210 μm	N/A	N/A	0.16	1223 K	72
EuCu_2_GeSe_4_	[EuSe_8_]	*Ama*2 (no. 40)	1.74	No signal	N/A	N/A	N/A	1073 K	75
EuCu_2_GeS_4_	[EuSe_8_]	*P*3_2_21 (no. 154)	2.32	No signal	N/A	N/A	N/A	1073 K	75
EuCu_2_SiS_4_	[EuS_8_]	*P*3_1_21 (no. 152)	2.36	No signal	N/A	N/A	N/A	1073 K	75
Dy_3_Si_0.85_Al_0.53_S_7_	[DyS_8_]	*P*6_3_ (no. 173)	2.03	1 × KTP@∼20 μm	N/A	N/A	N/A	1223 K	108
Dy_6_Al_2_SiS_14_	[DyS_8_]	*P*6_3_ (no. 173)	2.22	2 × KTP@∼20 μm	N/A	N/A	N/A	1223 K	108
Sm_3_Al_0.33_SiS_7_	[SmS_8_]	*P*6_3_ (no. 173)	2.26	0.3 × KDP@∼150 μm	N/A	N/A	N/A	1223 K	108
Y_6_ZnSi_2_S_14_	[YS_8_]	*P*6_3_ (no. 173)	2.38	0.84@∼20 μm	N/A	N/A	N/A	1223 K	108
La_3_GaGe_0.5_S_7_	[LaS_8_]	*P*6_3_ (no. 173)	2.54	4.8@74–106 μm	N/A	NPM	0.023	1223 K	82
Sm_3_GaGe_0.5_S_7_	[SmS_8_]	*P*6_3_ (no. 173)	2.50	Weak	N/A	N/A	N/A	1253 K	82
La_3_CuGeSe_7_	[LaSe_8_]	*P*6_3_ (no. 173)	2.00	0.3 × SiO_2_	N/A	N/A	N/A	1073 K	94
La_3_LiSnS_7_	[LaS_8_]	*P*6_3_ (no. 173)	2.40	1.2	2.5	PM	0.031	1073 K	126
Sm_3_LiSiS_7_	[SmS_8_]	*P*6_3_ (no. 173)	2.83	1.5 @88–105 μm	3.7	NPM	N/A	1373 K	124
La_3_LiGeS_7_	[LaS_8_]	*P*6_3_ (no. 173)	3.02	0.7	6.0	PM	0.025	1173 K	126
La_6_Ga_2_GeS_14_	[LaS_8_]	*P*6_3_ (no. 173)	2.54	4.8@74–106 μm	N/A	NPM	0.023	1223 K	82
La_6_In_2_GeS_14_	[LaS_8_]	*P*6_3_ (no. 173)	2.61	1.8@74–106 μm	N/A	NPM	0.007	1173 K	82
Y_6_Ga_2_GeS_14_	[YS_8_]	*P*6_3_ (no. 173)	2.30	Weak	3.0	NPM	N/A	1153 K	130
EuHgGeSe_4_	[EuSe_8_]	*Ama*2 (no. 40)	1.97	3.1	N/A	PM	0.31	1173 K	133
EuHgSnS_4_	[EuS_8_]	*Ama*2 (no. 40)	2.14	1.77	N/A	PM	0.33	1173 K	133
EuCdGeSe_4_	[EuSe_8_]	*Ama*2 (no. 40)	2.25	3.8	N/A	PM	0.18	1273 K	132
EuCdGeS_4_	[EuS_8_]	*Ama*2 (no. 40)	2.50	2.6	N/A	PM	0.18	1273 K	132
Eu_2_Ga_2_GeS_7_	[EuS_6_]	*P*4̄2_1_*m* (no. 113)	2.30	1.6@46–74 μm	N/A	NPM	0.098	1123 K	134
La_2_Ga_2_GeS_8_	[LaS_8_]	*Cmc*2_1_ (no. 36)	2.78	weak@46–74 μm	N/A	N/A	0.077	1373 K	134
Ba_2_InYSe_5_	[YSe_6_]	*Cmc*2_1_ (no. 36)	2.31	1 × AgGaSe_2_	N/A	N/A	N/A	1323 K	135
Ba_4_Sm_2_Cd_3_S_10_	[SmS_6_]	*Cmc*2_1_ (no. 36)	2.77	1.8@46–74 μm	14.3	N/A	0.044	1173 K	137
LaSrGa_3_S_7_	[LaS_8_]	*P*4̄2_1_*m* (no. 113)	2.92	1.3	5.0	PM	0.099	1273 K	139
LaCaGa_3_S_7_	[LaS_8_]	*P*4̄2_1_*m* (no. 113)	2.96	1.3	5.0	PM	0.134	1273 K	139
LaCaAl_3_S_7_	[LaS_8_]	*P*4̄2_1_*m* (no. 113)	3.76	0.8	9	PM	0.059	1473 K	138
LaSrAl_3_S_7_	[LaS_8_]	*P*4̄2_1_*m* (no. 113)	3.78	1.1	9	PM	0.077	1473 K	138
La_2_Ca_3_Sn_3_S_12_	[(La/Ca)S_7_]/[(La/Ca)S_9_]	*P*4̄2*m* (no. 189)	1.65	1.4@200–250 μm	N/A	NPM	0.008	1073 K	142
Sm_2_CaSn_3_S_12_	N/A	*P*4̄2*m* (no. 189)	1.66	1.2@200–250 μm	N/A	NPM	N/A	1073 K	142
Gd_2_Ca_3_Sn_3_S_12_	N/A	*P*4̄2*m* (no. 189)	1.63	1.0@200–250 μm	N/A	NPM	N/A	1073 K	142
La_2_Sr_3_Sn_3_S_12_	[(La/Ca)S_7_]/[(La/Ca)S_8_]	*Pmc*2_1_ (no. 26)	1.68	3.0	N/A	PM	0.086	1073 K	142
Sm_2_Sr_3_Sn_3_S_12_	N/A	*Pmc*2_1_ (no. 26)	1.71	2.5	N/A	PM	N/A	1073 K	142
Gd_2_Sr_3_Sn_3_S_12_	N/A	*Pmc*2_1_ (no. 26)	1.51	2.6	N/A	PM	N/A	1073 K	142
CeLiSiS_4_	[CeS_8_]	*Ama*2 (no. 40)	2.92	2.1	9	PM	0.054	1073 K	143
LaLiSiS_4_	[LaS_8_]	*Ama*2 (no. 40)	3.71	2.0	14	PM	0.033	1073 K	143
CsLaGeS_4_	[LaS_7_]	*P*2_1_2_1_2_1_ (no. 19)	3.60	0.5 × α-SiO_2_	N/A	N/A	N/A	1173 K	146
KYGeS_4_	[YS_7_]	*P*2_1_ (no. 4)	3.15	1.0	10	PM	0.12	1173 K	148
K_3_YP_2_S_8_	[YS_8_]	*P*2_1_ (no. 4)	3.37	1.4	7.0	PM	0.096	923 K	149
K_3_HoP_2_S_8_	[HoS_8_]	*P*2_1_ (no. 4)	N/A	1.1	3.0	PM	0.084	923 K	149
K_3_ErP_2_S_8_	[ErS_8_]	*P*2_1_ (no. 4)	N/A	1.2	2.5	PM	0.099	923 K	149

a Experimental value.

b Powder sample.

c PM = phase-matchability, NPM = nonphase-matchability.

d Theoretical value.

e N/A = not available.

f The maximum temperature of solid-state reaction.

**Table 2:** Summary of reported RE-based chalcogenides containing lone-pair-electron motifs^e^

Compound	[REQ_*n*_] polyhedra	Space group	*E*_g_ a (eV)	*d*_eff_ b (×AgGaS_2_)	LIDT^b^ (×AgGaS_2_)	NPM/PM^c^	Δ*n*^d^	Maximum temperature^f^	Ref.
Ce_8_Sb_2_S_15_	[CeS_7_]/[CeS_8_]	*I*4_1_*cd* (no. 110)	1.99	0.03	N/A	NPM	N/A	1223 K	163
La_8_Sb_2_S_15_	[LaS_7_]/[LaS_8_]	*I*4_1_*cd* (no. 110)	2.30	1.2@74–106 μm	N/A	NPM	N/A	1223 K	171
La_2_CuSbS_5_	[LaS_8_]/[LaS_10_]	*Ima*2 (no. 46)	2.06	0.5	6.7	PM	0.11	1273 K	154
La_4_InSbS_9_	[LaS_6_]/[LaS_7_]	*P*4_1_2_1_2 (no. 92)	2.07	1.5	N/A	PM	N/A	1223 K	156
Pr_4_InSbS_9_	[PrS_6_]/[PrS_7_]	*P*4_3_2_1_2 (no. 96)	2.09	No signal	N/A	N/A	N/A	1223 K	156
Nd_4_InSbS_9_	[NdS_6_]/[NdS_7_]	*P*4_3_2_1_2 (no. 96)	2.12	No signal	N/A	N/A	N/A	1223 K	156
Sm_4_InSbS_9_	[SmS_6_]/[SmS_7_]	*P*4_3_2_1_2 (no. 96)	2.13	0.75@150–210 μm	N/A	PM	N/A	1223 K	158
Y_4_GaSbS_9_	[YS_6_]/[YS_7_]	*Aba*2 (no. 41)	2.06	7.5 × α-SiO_2_@74–106 μm	N/A	NPM	N/A	1223 K	159
Sm_4_GaSbS_9_	[SmS_6_]/[SmS_7_]	*Aba*2 (no. 41)	2.23	3.8@46–74 μm	N/A	NPM	N/A	1223 K	157
Ho_4_GaSbS_9_	[HoS_6_]/[HoS_7_]	*Aba*2 (no. 41)	2.25	0.25@46–74 μm	N/A	N/A	N/A	1223 K	157
Gd_4_GaSbS_9_	[GdS_6_]/[GdS_7_]	*Aba*2 (no. 41)	2.41	0.8@46–74 μm	N/A	NPM	N/A	1223 K	157
Tb_4_GaSbS_9_	[TbS_6_]/[TbS_7_]	*Aba*2 (no. 41)	2.44	Weak	N/A	N/A	N/A	1223 K	157

a Experimental value.

b Powder sample.

c PM = phase-matchability, NPM = nonphase-matchability.

d Theoretical value.

e N/A = not available.

f The maximum temperature of solid-state reaction.

**Table 3:** A summary of reported RE-based chalcogenides containing [BS_3_] and [P_2_Q_6_] motifs^e^

Compound	[REQ_*n*_] polyhedra	Space group	*E*_g_ a (eV)	*d*_eff_ b (×AgGaS_2_)	LIDT (×AgGaS_2_)^b^	NPM/PM^c^	Δ*n*^d^	Maximum temperature^f^	Ref.
LaBS_3_	[LaS_9_]	*Pna*2_1_ (no. 33)	3.18	1.2	14	PM	0.143	1023 K	167
Ca_2_La(BS_3_)(SiS_4_)	[LaS_6_]	*P*6_3_*mc* (no. 186)	3.38	1.1	10	PM	0.149	1123 K	169
Ca_2_Ce(BS_3_)(SiS_4_)	[CeS_6_]	*P*6_3_*mc* (no. 186)	2.65	1.2	7	PM	0.149	1123 K	169
Ca_2_Gd(BS_3_)(SiS_4_)	[GdS_6_]	*P*6_3_*mc* (no. 186)	3.20	1.1	8	PM	0.126	1123 K	169
Eu_2_P_2_S_6_	[EuS_8_]	*Pc* (no. 7)	2.54	0.9	3.4	PM	0.074	1223 K	171
KSmP_2_Se_6_	[SmSe_8_]	*P*2_1_ (no. 4)	1.92	1.08	1.43	PM	N/A	1023 K	172
KTbP_2_Se_6_	[TbSe_8_]	*P*2_1_ (no. 4)	2.46	0.35	2.29	PM	N/A	1023 K	172
KGdP_2_Se_6_	[GdSe_8_]	*P*2_1_ (no. 4)	2.53	0.44	4.33	PM	0.081	1023 K	172

a Experimental value.

b Powder sample.

c PM = phase-matchability, NPM = nonphase-matchability.

d Theoretical value.

e N/A = not available.

f The maximum temperature of solid-state reaction.

**Table 4:** A summary of reported RE-based chalcohalides and oxychalcogenide^e^

Compound	[REQ_*n*_] polyhedra	Space group	*E*_g_ (eV)^a^	*d*_eff_ (×AgGaS_2_)^b^	LIDT ( ×AgGaS_2_)^b^	NPM/PM^c^	Δ*n*^d^	Maximum temperature^f^	Ref.
La_6_Cd_0.75_Ga_2_Se_11.5_Cl_2.5_	[LaSe_7_]	*P*6_3_ (no. 173)	1.65	0.1@43–75 μm	N/A	NPM	N/A	1173 K	173
La_6_Cd_0.75_Ga_2_S_11.5_Cl_2.5_	[LaS_7_]	*P*6_3_ (no. 173)	2.28	0.8@43–75 μm	18.6	NPM	N/A	1173 K	173
La_3_AsS_5_Br_2_	[LaS_7_]	*Cc* (no. 9)	2.90	0.23	N/A	N/A	N/A	1123 K	174
Eu_4.5_(B_5_O_9_)_2_SI	[EuO_5_S_2_I]/[EuO_5_S_2_I]/[EuO_4_S_2_]	*Pnn*2 (no. 34)	1.99	0.5	15	PM	N/A	1223 K	176
(K_3_I)[SmB_12_(GaS_4_)_3_]	[SmS_6_]	*P*6_3_22 (no. 182)	2.35	0.3 × KTP	N/A	N/A	N/A	1223 K	177
[La_3_OSCl_2_][SbS_3_]	[(Cl/S)La_3_]	*P*6_3_*mc* (no. 186)	2.50	0.7	N/A	N/A	0.269	1223 K	178
YSeBO_2_	[YO_3_Se_4_]^11−^	*Cmc*2_1_ (no. 36)	3.45	0.0054	N/A	PM	N/A	1223 K	180
Eu_3_GeOS_4_	[EuOS_6_]	*Pca*2_1_ (no. 29)	2.05	0.24@110–150 μm	8.86	NPM	0.019	1148 K	181
Sm_3_NbS_3_O_4_	[SmS_8_]	*Pna*2_1_ (no. 33)	2.68	0.3	12.5	PM	N/A	1273 K	185
Gd_3_NbS_3_O_4_	[GdS_8_]	*Pna*2_1_ (no. 33)	2.74	0.4	4.5	PM	N/A	1273 K	185
Nd_3_[Ga_3_O_3_S_3_][Ge_2_O_7_]	[NdO_6_S_2_]	*P*4̄2*c* (No. 190)	4.35	0.8	23	PM	0.091	1273 K	186
Eu_2_MnGe_2_OS_6_	[EuOS_7_]	*P*4̄2_1_*m* (no. 113)	2.40	0.3	8.3	PM	0.13	1173 K	187
Eu_2_FeGe_2_OS_6_	[EuOS_7_]	*P*4̄2_1_*m* (no. 113)	2.11	0.3	2.8	PM	N/A	1173 K	187
Eu_2_CoGe_2_OS_6_	[EuOS_7_]	*P*4̄2_1_*m* (no. 113)	2.14	0.5	3.2	PM	N/A	1173 K	187
LaSrGa_3_S_6_O	[LaS_7_O]	*P*4̄2_1_*m* (no. 113)	3.21	1.0	14	PM	0.138	1273 K	139
LaCaGa_3_S_6_O	[LaS_7_O]	*P*4̄2_1_*m* (no. 113)	3.27	0.9	14	PM	0.163	1273 K	139

a Experimental value.

b Powder sample.

c PM = phase-matchability, NPM = nonphase-matchability.

d Theoretical value.

e N/A = not available.

f The maximum temperature of solid-state reaction.

(1) Due to the Gd discontinuity effect, there is a slight variation in the ionic radius of lanthanide elements, which leads to differences in their chemical and physical properties. Therefore, based on differences in electronic configuration, as well as physical and chemical properties, the elements preceding Gd are classified as light RE elements or cerium-group elements (La–Eu), while the remaining elements are classified as heavy RE elements (Gd–Lu, Y, and Sc). As shown in Scheme 1, light RE-based chalcogenide compounds have greater appeal than heavy ones. Based on the classification of light and heavy RE elements, an analysis of these compounds reveals that the probability of obtaining NCS compounds through the synthesis of light RE elements is 47.9% (excluding the radioactive *Pm* element), which is twice the average of heavy RE elements (20.0%). Furthermore, the NLO-active compounds in the light RE elements group account for a remarkable 70.7% of the total, which is also double the percentage found in the heavy group (29.3%). Of course, the price difference between light and heavy RE elements will also impact the synthesis and amount of research into the compounds. However, a more profound reason might be that the synthesis of light RE elements is more conducive to obtaining NCS structures and exhibiting NLO activity. Moreover, by statistically analysing the NCS compounds of each RE element and specifically examining those with NLO activity, it is observed that the top three elements are Eu (88.2%), La (38.2%), and Sm (29.7%), which all belong to the light RE category. Additionally, the percentage of NLO-active materials among Eu-based NCS materials is remarkably high, reaching 88.2%, surpassing the second-ranked La (38.2%). This demonstrates that among the 16 elements (excluding the radioactive *Pm*), Eu-based compounds stand out as promising candidates for IR-NLO materials with excellent potential.

(2) These compounds can be divided into four categories based upon the number of elements: ternary (9, 10.6%), quaternary (62, 72.9%), quinary (13, 15.3%), and hexanary (1, 1.2%). Furthermore, in terms of the dimensionality of the crystal structure, there are 6 (7.1%) 0D compounds, 1 (1.2%) 1D compound, 22 2D (25.9%) compounds, 42 (49.4%) 3D compounds, and 14 (16.5%) MD compounds. Additionally, based on the crystal systems, they can be divided into five categories: monoclinic (12, 14.1%), orthorhombic (31, 36.5%), tetragonal (16, 18.8%), trigonal (2, 2.4%), and hexagonal (24, 28.2%). Analysis of the space group distribution revealed that the top three space groups are *P*6_3_ (No.173, 15 examples), *Cmc*2_1_ (No.36, 11 examples), and *P*4̄2_1_*m* (no. 113, 10 examples) (see Scheme 2 for details).

(3) The RE-based asymmetric building motifs that have been reported currently exhibit two different coordination modes. These include the single-anion coordination mode, which comprises [REQ_*n*_] (*n* = 6–10), and the heteroanion coordination mode, which consists mainly of [REQ_2_O_4_], [REQ_4_O_3_], [REQ_6_O], [REQ_7_O], [REQ_2_O_6_], [REQ_4_OX_3_], and [REQ_2_O_5_X] (Scheme 3). It is important to note that the structures of these asymmetric building motifs determine their performance. Consequently, RE-based materials composed of these different asymmetric building motifs exhibit distinct IR-NLO properties. In Scheme 4, a comparison of the *d*_eff_ and *E*_g_ of RE-based IR-NLO materials is displayed. La_6_Ga_2_GeS_14_ has the largest *d*_eff_ of 4.8 × AgGaS_2_ at a particle size of 74–106 μm among all the RE-based chalcogenide IR-NLO materials, but it has a relatively small *E*_g_ of 2.54 eV. Nd_3_[Ga_3_O_3_S_3_][Ge_2_O_7_] has the largest *E*_g_ of 4.35 eV, while it has a relatively small *d*_eff_ of 0.8 × AgGaS_2_ at a particle size of 150–210 μm. The “performance-balanced area” indicates the potential materials whose *d*_eff_ and *E*_g_ are both higher than those of AgGaS_2_. In this performance-balanced area, there are 11 PM chalcogenides, but only 1 PM oxychalcogenide and no chalcohalides, which demonstrates the outstanding performance of RE-based chalcogenides. The exploration of oxychalcogenides and chalcohalides should be continued to enable a more in-depth analysis of their structural chemistry and understanding the origin of their properties through additional experimental results.

(4) For the synthesis method of RE-based IR-NLO compounds, the common method is high-temperature solid-state synthesis. Additionally, the introduction of RE elements not only involves the use of elemental RE, but also employs the reactive flux and boron–chalcogen mixture methods. The boron–chalcogen mixture method uses boron and RE oxides to remove oxygen *in situ* from rare earth oxides during the reaction, allowing the participation of RE elements. The emergence of this method enriches the selection of reaction pathways.

Currently, out of over 400 RE-based compounds, fewer than 100 demonstrate NLO properties. With decades of accumulated knowledge, our predecessors have laid a substantial foundation for us. Now is the opportune moment to strategically delve into synthesizing and exploring the underlying mechanisms, cultivating the soil to harvest successful outcomes. For this, the following outlook is provided:

(1) Further investigation into the mechanisms of RE-based polyhedra is needed. Currently, a significant question remains unanswered in the realm of RE-based compounds: why do only certain compounds of RE elements exhibit nonlinear effects in isomeric compounds? The differences among RE cations go beyond simple considerations of atomic radius; the underlying reasons should be attributed to variations in their electronic structures. Previous researchers have explored and synthesized numerous compounds in this field. In-depth studies of the electronic structures of different cations can help to uncover the answer to this question. Additionally, the contribution of the 5d and 4f empty orbitals of RE cations plays a crucial role in the conductive bands. By delving into the electronic structures of various anions and selectively substituting RE cations and anions with different 4f and 5d electronic structures based on their distinct structures, blind synthesis of RE-based compounds can be avoided, and hidden treasures within the accumulated knowledge can be uncovered. In contemporary research, the continual progress in theoretical calculations and machine learning plays a pivotal role in the exploration and design of novel materials. When synthesizing RE-based chalcogenides, the substitutability of RE sites with various RE elements is commonly considered, yet synthesis endeavours are often time-consuming. Consequently, upon successfully synthesizing a particular compound, it becomes important to validate, through theoretical calculations, the generalizability of the synthesis method to other RE elements. Additionally, when investigating isostructural compounds, a judicious approach involves preliminary theoretical analyses to identify RE elements that may exhibit superior performance, guiding subsequent synthesis explorations.

(2) According to Pauling's third and fourth rules, in systems containing more than one type of cation, polyhedra with high oxidation states and low coordination numbers tend to be connected through sharing corners or remain unconnected. The two functional motifs [PS_4_] and [SiS_4_], which are beneficial in terms of *E*_g_, can be taken as examples. Due to the high positive charge and low coordination of the central cations, self-aggregation into high-dimensional frameworks becomes challenging under electrostatic rules. Therefore, a common approach for stabilization is to connect them with other polyhedra possessing central cations with lower positive charges and higher coordination. When alkali earth metals or alkali metals bond with these functional motifs, the resulting bonds are primarily ionic in nature. One characteristic of ionic bonds is isotropy, which makes it difficult to restrict the orientation of functional motifs and thus facilitates the formation of a thermodynamically stable centrosymmetric phase. Additionally, the isotropic nature of these bonds leads to low optical anisotropy. The bonding between RE^*n*+^ and S^2−^ is complex, primarily due to the complexity of their respective electronic structures. Furthermore, as different RE^*n*+^ engage in bonding, the variability in their individual electronic structures contributes to distinct electron density of states within the system. As a result, this diversity leads to varying responses to external light stimuli. The presence of high-coordination RE cations acts as a binder between polyhedra with high oxidation states and coordination numbers. In these systems, RE cations not only play a role as a structural binder, but also contribute to providing nonlinear optical responses. Therefore, re-examining the structures of different compounds in the database reveals that introducing RE cations to connect previously disordered functional units may have a rejuvenating effect of “old wood meets spring”, enhancing performance while adjusting the structure.

(3) The growth of large crystals is necessary. Currently, the major RE-based compounds are tested in the powder state, but the real working state of crystal materials is the single-crystal state. Consequently, in order to satisfy the demands of test accuracy, further test results are needed in the large single-crystal state, which is also a prerequisite for evaluating the commercialization of NLO materials.

## Data availability

There is no experimental or computational data associated with this article.

## Author contributions

Ping Feng: investigation, writing – original draft. Jia-Xiang Zhang: investigation, writing – original draft. Mao-Yin Ran: investigation, formal analysis. Xin-Tao Wu: conceptualization, formal analysis. Hua Lin: supervision, conceptualization, writing – review & editing. Qi-Long Zhu: supervision, writing – review & editing.

## Conflicts of interest

There are no conflicts to declare.

## Supplementary Material
